# Author Correction: Pan-cancer multi-omic model of LINE-1 activity reveals locus heterogeneity of retrotransposition efficiency

**DOI:** 10.1038/s41467-025-58288-2

**Published:** 2025-03-24

**Authors:** Alexander Solovyov, Julie M. Behr, David Hoyos, Eric Banks, Alexander W. Drong, Bryan Thornlow, Jimmy Z. Zhong, Enrique Garcia-Rivera, Wilson McKerrow, Chong Chu, Cedric Arisdakessian, Dennis M. Zaller, Junne Kamihara, Liyang Diao, Menachem Fromer, Benjamin D. Greenbaum

**Affiliations:** 1 Halvorsen Center for Computational Oncology, Department of Epidemiology and Biostatistics, Memorial Sloan Kettering Cancer Center, New York, NY USA; 2 ROME Therapeutics, Inc., Boston, MA USA; 3 Acorn Biosciences, Cambridge, MA USA; 4 Division of Hematology/Oncology, Boston Children’s Hospital, Boston, MA USA; 5 Department of Pediatric Oncology, Dana-Farber Cancer Institute, Harvard Medical School, Boston, MA USA; 6 Division of Population Sciences, Dana-Farber Cancer Institute, Harvard Medical School, Boston, MA USA; 7 Physiology, Biophysics & Systems Biology, Weill Cornell Medical College, New York, NY USA

**Keywords:** Cancer, Computational biology and bioinformatics

Correction to: *Nature Communications* 10.1038/s41467-025-57271-1, published online 28 February 2025

In the Acknowledgements section, the name ‘Agnel Sfeir’ was incorrectly given as ‘Agnel Safir’. In addition, the funding from ‘Halvorsen Center for Computational Oncology at Memorial Sloan Kettering Cancer Center’ was omitted. The original article has been corrected.

