# Supplementary material for: Pan-cancer multi-omic model of LINE-1 activity reveals locus heterogeneity of retrotransposition efficiency
Source: Nat Commun. 2025 Feb 28;16:2049. doi: 10.1038/s41467-025-57271-1 (PMC11871128; doi:10.1038/s41467-025-57271-1)
Supplement: Supplementary file 1 — Supplementary Information [file 41467_2025_57271_MOESM1_ESM.pdf]

Pan-cancer multi-omic model of LINE-1 activity reveals locus heterogeneity of retrotransposition efficiency

Alexander Solovyov[1,*,†], Julie M. Behr[2*], David Hoyos[1], Eric Banks[2,3], Alexander W. Drong[2], Bryan Thornlow[2], Jimmy Z. Zhong[2], Enrique Garcia-Rivera[2], Wilson McKerrow[2], Chong Chu[2], Cedric Arisdakessian[2], Dennis M. Zaller[2], Junne Kamihara[4,5,6], Liyang Diao[2,#], Menachem Fromer[2,#], Benjamin D. Greenbaum[1,7,#,†]

[1]Halvorsen Center for Computational Oncology, Department of Epidemiology and Biostatistics, Memorial Sloan Kettering Cancer Center, Memorial Sloan Kettering Cancer Center, New York, NY, USA
[2]ROME Therapeutics, Inc., Boston, MA, USA
[3]Acorn Biosciences, Cambridge, MA, USA
[4]Division of Hematology/Oncology, Boston Children's Hospital, Boston, MA, USA
[5]Department of Pediatric Oncology, Dana-Farber Cancer Institute, Harvard Medical School, Boston, MA, USA
[6]Division of Population Sciences, Dana-Farber Cancer Institute, Harvard Medical School, Boston, MA, USA
[7]Physiology, Biophysics & Systems Biology, Weill Cornell Medical College, New York, NY, USA

*These authors contributed equally to this work
[#]These authors jointly supervised this work
†Corresponding authors: solovyova@mskcc.org, greenbab@mskcc.org

The Supplementary Information consistent of a Supplementary Methods, Supplementary Figures, and a Data Dictionary and description for the Supplementary Data, which are included as set of downloadable tables.

Supplementary Methods

L1 retrotransposition detection from short-read whole genome sequencing data

Despite its frequent reintegration in cancer genomes 11, measuring L1 retrotransposition from short-read sequencing data to accurately quantify its movement in cancer poses technical challenges due to the many genomic copies of highly similar L1 sequences, as well as different modes of the retrotransposition process (Fig. 1a-d), making it difficult to disambiguate the source of L1-containing sequencing reads. While methods have been developed for detecting somatic retrotransposition events, we set out to build a first principles approach that accounts for the specific retrotransposition cycle of L1 (Fig. 1). The resulting “TotalReCall” method relies on two signals that arise when aligning sequencing reads to the reference genome (Fig. 1g): (i) reads that span the retrotransposon insertion site breakpoint will result in chimeric reads which contain portions of both the insertion site sequence from the human genome as well as the inserted L1 sequence, and lead to “soft clipped” alignments, and (ii) paired-end reads that arise from fragments spanning the inserted L1 sequence will often have one read map near the insertion site, but the other read map to one of the many L1 sequences elsewhere in the genome, even to a different chromosome or a distant site on the same chromosome, resulting in “discordant read pairs”.

We use the clipped reads as the primary signal because of their higher specificity: the last mapping point as well as the clipped sequence are the same (up to sequencing errors) for all the clipped reads supporting the same breakpoint. Interpretation of these types of signals arises from the mechanistic biochemistry of the life cycle of retrotransposons. Class I transposable elements replicate via a reverse transcribed RNA intermediate (“copy-and-paste”) in a process known as retrotransposition, whereas class II elements replicate via a DNA intermediate (“cut-and-paste”).

Further subdividing based on distinct mechanisms of mobility, there are three major categories of transposable elements: class I LTR retrotransposons (including endogenous retroviruses), class I non-LTR retrotransposons, and class II DNA transposons[6]. Among the transposable elements, only some non-LTR retrotransposons are known to be able to move within the genome. L1 elements are capable of autonomous retrotransposition, and SINE (e.g., Alu) and SVA elements are "parasites" that rely on the L1 machinery for mobility.

The difference between the mechanisms utilized by LTR and non-LTR retrotransposons is that the LTR retrotransposons first synthesize the double stranded DNA from RNA transcript in a complex process involving the long terminal repeats (LTRs) and then integrate that sequence into the host genome. On the other hand, enzymes of non-LTR retrotransposons (LINE elements) reverse transcribe their RNA directly into the genome using the so-called target-primed reverse transcription (TPRT) process[7].

Unlike the LTR retrotransposons, which always integrate the complete provirus with the LTRs into the genome (though, sometimes, the internal provirus part may be removed by homologous recombination leaving a solo LTR), reverse transcription of a non-LTR retrotransposon may be terminated prematurely resulting in a partial integration. Apart from such a 5' truncation, another common structural variant of L1 is inversion resulting from twin priming[8] and simultaneous reverse transcription of the same L1 transcript into both strands of the genome (Fig. 1d)[8,9]. In addition, it is not uncommon for there to be 3' transductions, where during L1 RNA expression, the polymerase overruns the poly(A) signal at the 3' end of the L1 element resulting in transcription and retrotransposition of some genomic sequence downstream of the 3' end of the source L1 element in addition to the L1 element itself [10,11]. In the TotalReCall method developed here, we focus on detecting 5' truncation and L1 inversion. We believe that the analysis of even rarer and more complex structural variations arising from L1 retrotransposons (e.g., instances with more than one template switching during the reverse transcription process, which would result in a series of inversions) is better left for long-read data.

We thus designed the TotalReCall algorithm (Fig. 1) to detect both "canonical" retrotransposition events and "inversion-containing" retrotranspositions. In particular, "single-stranded" retrotransposition (Fig. 1c) leads to a canonical retrotransposition with a single segment of L1 inserted into the genome (Fig. 1e), with the two ends of the insertion detectable in the clipped short-reads (Fig. 1g). On the other hand, a twin priming retrotransposition event (Fig. 1d), where each strand of the target site genomic DNA incorporates reverse transcribed sequence of a single L1 mRNA, leads to an inversion-containing retrotransposition with a DNA insertion that contains part of a L1 sequence and another part of it that is inverted, i.e., contains reverse complementary L1 sequence, in an adjacent position in the genome (Fig. 1f), which will be reflected in the short-reads data (Fig. 1g).

To identify the breakpoints of the L1 insertion event, TotalReCall collects the clipped sequences of the reads in the vicinity, sort them by length in decreasing order and cluster in a cd-hit-like[12,13] way: take each new sequence and align it against the existing representatives; if a match is found, assign the sequence to the corresponding cluster, otherwise designate it as a new representative. We group the reads representing the "left" (clipped on the 3' end w.r.t. the reference genome) and the "right" (clipped on the 5' end w.r.t. the reference genome) breakpoints. The longest clipped sequence in the cluster serves as the representative of the breakpoint. If we orient the clipped sequence so that the clipping point is at its 5' end, a canonical L1 retrotransposition is characterized by one breakpoint with the poly(T) sequence (regardless of whether a 3' transduction is present) and the other breakpoint with a sequence mapping to the positive strand of the L1 sequence. The coordinate of the alignment of the clipped sequence at the breakpoint to

the L1 sequence can be used to infer the length of the transposon (without the 3' transduction, if one exists). An inversion-containing L1 retrotransposition is characterized by one breakpoint bearing the poly(T) sequence (regardless of whether a 3' transduction is present) and the other breakpoint with a sequence mapping to the negative strand of the L1 sequence. We increased sensitivity by utilizing the supplementary alignments and inferring the (usually hard-) clipped part of their sequence from the matching primary alignments. The use of such supplementary alignments is highly desirable since they provide a longer clipped sequence, which can in turn be aligned to the L1 sequence more specifically.

TotalReCall also identifies reads that belong to discordant read pairs and map near the breakpoints and use their mates as a secondary signal to determine the confidence of the call. Clipped sequences, as well as sequences of the reads belonging to discordant read pairs, were aligned to the L1 consensus sequence using LAST aligner[14]. The consensus sequence of the L1 transposon was constructed by merging the three DFAM[15] sequences for its 3', 5' ends and ORF2 subdomain (DF0000225, DF0000226, DF0000316). We resolved the ambiguous characters in the DFAM consensus sequences using the alignment of the 146 intact L1 sequences from L1base[16].

To identify somatic L1 retrotranspositions (i.e., in our typical use case of tumor-specific insertions), TotalReCall checked each call in the case sample for the presence in its control sample of the signal of a clipped read with a matching sequence or a breakpoint, retaining as "somatic" (i.e., case-specific) only calls without such signal in the control. In addition, we implemented filters for low complexity and regions with large numbers of alignment artifacts ("high entropy regions"), e.g., regions having too many clipped reads with distinct sequence and low complexity regions where genomic sequence matches clipped sequence.

We have reviewed 500 randomly selected screenshots of LINE-1 calls from TCGA-LUSC and identified the loci with a clear signal: clipped reads at the 5' and the 3' end and possibly some discordant read pairs. Then we trained a naive Bayesian classifier as implemented in scikit-learn using these data and applied it to all TCGA and GIAB calls. We used the following independent variables to train the classifier:

1. bitscore for the local alignment of the clipped sequence at the 5' end breakpoint to L1,
2. bitscore for the localalignment of the clipped sequence at the 3' end breakpoint to the poly(A) possibly followed by the 3' end of L1,
3. length of the unaligned end at the 5' end of the clipped sequence at the 5' end breakpoint,
4. length of the unaligned end at the 3' end of the clipped sequence at the 5' end breakpoint,
5. number of clipped reads with a matching sequence at the 5' end breakpoint,
6. whether low complexity filter is triggered (binary variable),
7. normalized number of reads not properly mapped as a pair whose mates map to L1 – separate numbers for the 3' end and the 5' end as well as reads which are clipped at the breakpoint with a matching sequence or not (4 numbers total).

It is worth mentioning that the short-read data possess inherent limitations, for example, difficulty identifying 3' transductions. Long such transductions may be identified via discordant read pairs whose mates map downstream of source L1 element. At the same time, this method will fail to identify short 3' transductions. Some other shortcomings are the ambiguity of pairing between the left/right breakpoints if multiple ones are present in the same region as well as low mappability of some genomic regions (in particular, pericentromeric regions).

Non-reference LINE-1 insertions present in both case and control samples

TotalRecall was used to determine the set of “pseudo” germline retrotranspositions, e.g., those RT calls which are present in a tumor sample, and which also have some evidence at the alignment level for being present in the paired normal sample. Only TotalRecall was used, as xTea was not run in germline mode.

As described in the previous section, TotalRecall uses the case sample to make a call, then checks this against the control sample. If there is at least one clipped read with the matching sequence at the matching location in the control alignment file, the call is filtered out—e.g., the call is classified as non-somatic. Starting with this set of non-somatic retrotranspositions, we further required at least three clipped reads to be present at both the 3’ breakpoint and the 5’ breakpoint, filtered out L1 insertions shorter than 50bp, and filtered out possible false positives in low complexity regions, to ensure a stringent threshold for determining the “pseudo” germline set.

These calls need further validation using orthogonal technology, for example long-read sequencing. One of the concerns with the calling of “pseudo”-germline retrotranspositions is the possibility of sequencing artifacts in both tumor and normal samples which would not affect the somatic calls but can lead to false positive germline calls. Possible false negatives may also be caused by, for example, deletion of a germline non-reference LINE-1 element in the tumor. Calls were uploaded to dbGAP as described in the Data Availability statement.

Validating retrotransposition detection by TotalReCall, xTea, and TraFiC-mem using long-reads

For the validation using the Genome in a Bottle dataset we ran TotalReCall, xTea and TraFiC-mem using different members of the trio as “case” (downsampled to 80x) and “control” (downsampled to 35x), resulting in 6 pairwise comparisons. We checked the calls using an orthogonal technology (Oxford Nanopore long-reads aligned to hg19). We reviewed alignments of the long-reads in IGV checking for the presence of insertions and possibly clipped reads at the loci identified using short reads. In addition to that we extracted representative insertion sequences at the relevant loci from the alignment of the long-reads using pysam library. When there were many reads with insertions at the same locus, we picked representative insertion sequences with the highest mean quality. To compute the mean quality, we first zeroed out all base qualities which were smaller than 15 and then computed the mean base quality for the sequence of the insertion (keeping bases where qualities were zeroed out).

We ran BLASTn using the consensus L1 sequence as the subject and the representative insertion sequences as the query in order to determine the presence of the inversion (See Supplementary Data 5 for the validation of the inversion calls where TotalReCall and xTea disagree).

For the computation of the sensitivity of the methods using the short reads we composed the full long-read L1 call set (“ground truth”) as follows. We ran tldr[1] on the aligned Nanopore reads and selected non-reference L1 elements present in one or two members of the trio. We added the L1 insertions identified using short read data and verified to be supported by the long-read data as described above to the true call set.

Quantification of LINE-1 expression at the locus level

To validate that L1EM can be used to identify the specific LINE-1 loci expressed in a given sample, we simulated reads for each intact reference locus one at a time and then tested whether L1EM matched that simulated expression back to the correct locus.

Polyester (cite pmid: 25926345) was used to simulated 50, 100, 200, 500, 1000, or 5000 reads from each intact LINE-1 locus, one at a time, using default settings. Simulated reads in fasta format were converted to fastq using seqtk to add a constant quality string of ‘A’. The resulting fastqs were then processed identically to reads from the TCGA samples.

We found that L1EM typically assigns about 90% of the expression back to the correct locus, indicating that it is accurate enough to identify the loci expressed in a given tumor sample. We similarly tested TElocal (https://github.com/mhammell-laboratory/TElocal) and found that while TElocal as ~80% accurate, it did not perform as well as L1EM in most simulations. See Supplementary Fig. 2.

Locus clustering by RNA expression and RT events

For the individual clustering, based on one of RNA means or TRT means, the clustermap function of the seaborn library in Python was used. We defined clusters within each dendrogram using the fcluster function from the scipy module cluster.hierarchy. Briefly, this method computes a distance matrix (here, locus-locus Euclidean distances) to define hierarchical clusters with the UPGMA algorithm (method=’average’). Clusters were produced using an empirical distance cutoff for each feature (2 for RNA and 0.1 for TRTs).

In the tables below, we give the number of loci that fall into each of the RNA and TRT clusters. In particular, we highlight RNA cluster 3 and TRT cluster 1, both of which contain the largest number of loci, and which correspond to loci with the lowest RNA expression and TRT activity, respectively. These two clusters are highlighted for readability. We then provide how these loci are then combined into the final meta-cluster, which is presented in Fig. 5. For example, there are N=4 loci which fall into the RNA cluster 3, TRT cluster 2, and whose average TRTs across tumor types does not fall within the lowest histogram bin; these N=4 loci are clustered together in Fig. 5.

Number of loci per cluster in RNA and TRT clusterings

RNA CLUSTER	N
**3**	1470
1	4
2	3
5	2
6	1
7	1
4	1

TRT CLUSTER	N
**1**	1472
2	4
3	2
4	1
7	1
5	1
6	1

Number of loci per cluster in combined clustering

Giving also cluster membership of individual clusterings as above. The column “TRT LOWEST BIN” indicates whether the cluster contains loci that fall into the histogram bin of lowest average TRTs across tumor types (fewer than 0.0018 log2, QC-adjusted TRTs per sample).

RNA CLUSTER	TRT CLUSTER	TRT LOWEST BIN	N
**3**	**1**	True	1448
**3**	**1**	False	13
**3**	2	False	4
1	**1**	True	4
2	**1**	True	3

**3**	3	False	2
5	**1**	True	2
**3**	4	False	1
6	**1**	True	1
7	7	False	1
**3**	5	False	1
**3**	6	False	1
4	**1**	True	1

Simulating p53 regulation of L1

A Jupyter notebook (written in R) recreating the simulations of uni-modal p53 regulation is available in the GitHub repository accompanying this manuscript. The reverse partial correlation model, shown in Supplementary Fig. 10, was evaluated to test whether our dataset could support p53 regulation of L1 RT alone, and the apparent association between p53 and L1 RNA is spurious. Because p53 is correlated with L1 RT and L1 RNA is strongly correlated with L1 RT, correlation between p53 and L1 RNA could arise even in the absence of a transcriptionally regulatory relationship. The mediation model evaluated in Fig. 7 cannot determine whether the placement of independent, mediating, and dependent variables is inappropriate. We therefore sought to simulate scenarios in which p53 only regulates one phase of the L1 life cycle, whether influencing L1 RNA expression or killing cells with L1 RT, in order to evaluate the likelihood of a false positive result of our model in suggesting dual regulation.

We defined a simple system using binary p53 mutations (i.e. p53 is either WT or mutated). For every simulation, we define a likelihood of a p53 mutation occurring, the mean L1 RNA level (TPM) in p53 WT and mutant cells, the mean efficiency of RTs per TPM, the likelihood of WT p53 killing a cell with RTs, and the likelihood of mutant p53 killing a cell with RTs. For a given likelihood of p53 mutation occurring, a dataset of cells is initialized as either p53 mutant or WT based on random number generation. L1 RNA is sampled from a gamma distribution for each cell, based on the mean RNA level for the given p53 mutation status. L1 RT is sampled from a Poisson distribution where λ = the given RNA level · the defined mean efficiency. Each cell either lives or is killed by p53 based on comparing the likelihood of the given p53 mutation status killing the cell to a random number generator. The likelihood of p53 killing the cell is given by:

$$1 - (1 - p53\ kill\ probability_{mutation\ status})^{RT\ burden} \mid p53\ mutation\ status$$

All cells killed by p53 are removed from consideration. For the surviving cells, a mediation model (including the reverse partial correlation model) is evaluated.

In the first set of simulations, we assumed p53 only regulates L1 RT by killing cells with a non-0 RT burden and has no direct regulatory impact on L1 RNA. We therefore set mean L1 RNA to be equal in p53 mutant and WT simulated cells. We ran 10 replicates each of a combination of input values:

- Likelihood of a p53 mutation arising ranging from 1-6%
- Mean L1 RNA ranging from 10 to 45 TPM, at increments of 5 TPM
- Mean RTs per TPM ranging from 0.1 to 0.5, at increments of 0.1 RT/TPM
- Likelihood of WT p53 killing a cell with RT ranging from 60-100%, at increments of 10%
- Likelihood of Mutant p53 killing a cell with RT = x · likelihood of WT p53 killing a cell, for x in [0, 0.25, 0.5]
-

We used this set of simulations to evaluate the likelihood of a false positive result of p53 regulating L1 RNA, and therefore considered the coefficient assigned to p53 in the reverse model:

$$RNA \sim p53 + RT$$

Although our simulation input values were comparable to what we observe in real data, we used the t-value of the regression coefficient fit to compare across simulations and against our real data. In the mediation model using a binary value to represent p53 mutation (i.e. assign a value of 1 if p53 is mutated in a given tumor and 0 if it is not), the reverse model p53 coefficient has a t-value of 5.9. Based on our simulations, a t-value of 5.9 has an empirical likelihood $p = 2 \times 10^{-4}$ of occurring if p53 only regulates L1 RT and not L1 RNA. Although our mediation model from observational data cannot prove causation, we believe the data is inconsistent with a system in which p53 does not regulate L1 RNA beyond the extent of its regulation of L1 RT.

In the second set of simulations, we assumed p53 only regulates L1 RNA transcription, and does not kill cells in response to L1 RT. We therefore set the likelihood of p53 WT and mutant killing a cell to 0. We ran 10 replicates each of combinations of the following input values:

- Likelihood of a p53 mutation arising ranging from 20-60%, at increments of 10%
- Mean L1 RNA in p53 WT samples ranging from 10 to 55 TPM, at increments of 5 TPM
- Mean L1 RNA in p53 mutant samples ranging from 10 to 55 TPM, at increments of 5 TPM, provided that the mean L1 RNA in mutant p53 samples > mean L1 RNA in WT p53 samples for every simulation
- Mean RTs per TPM ranging from 0.1 to 0.5, at increments of 0.1 RT/TPM

In this set of simulations, we were testing the likelihood of a false positive association between L1 RT and p53 when controlling for L1 RNA, and therefore considered the coefficient assigned to p53 in the partial correlation model, which makes up the "unmediated" portion of the overall fit:

$$RT \sim p53 + RNA$$

We again compared the resulting t-values of the simulated regression coefficient fits to the t-value of the regression coefficient fit for our real data. In the mediation model using binary p53 mutations, this p53 coefficient has a t-value of 9.7. None of the empirical t-values (from 11,250 tests) were this high, meaning an empirical likelihood $p < 10^{-4}$ of observing this coefficient if p53 only regulates L1 RNA but does not independently affect L1 RT. Because L1 RNA precedes L1 RT, this effect was also easier to directly see in our data, by stratifying tumors based on L1 RNA levels and comparing L1 RT in p53 mutant vs WT tumors, as shown in Fig. 7d. Although our observational study cannot reveal mechanisms, we feel confident that our data support multiple modes of regulation and would be inconsistent with a system in which (through any mechanisms) p53 only impacts L1 RNA or L1 RT but not both.

Stratifying dataset by clinical annotations

Clinical annotations of samples, including age at diagnosis (reported as days to birth from initial pathologic diagnosis date) and overall survival, were collected by the TCGA Research Network and aggregated into a GitHub repository by J. Creed and T. Gerke (https://github.com/GerkeLab/TCGAclinical/raw/master/data/cBioportal_data.tsv, accessed 31 July 2023 – 29 March 2024, commit 8a95a11 updated 12 July 2019). Age at diagnosis was converted to approximate age in years, and then rounded down to the nearest decade. All tumor samples with RNA-seq data and an age at diagnosis were included in Supplementary Fig. 21 a,c, N = 8,367. All tumor samples with WGS data and an age at diagnosis annotation were included in Supplementary Fig. 21 b,d, N = 4,286.

For survival analyses, all tumor samples with WGS data and annotations for both "Overall Survival (Months)" and "Progress Free Survival (Months)" were used. Tumor samples were stratified based on whether they had any RT (i.e. non-zero RT burden) or none. In Supplementary Fig. 16a, tumor samples were additionally stratified by whether the age of the patient at diagnosis was over or under 50 years (N = 4,276). In Supplementary Fig. 16b, tumor samples were additionally stratified by whether or not they had a p53 mutation, as annotated by cBioPortal (N = 4,428).

Overall survival is plotted using the KaplanMeierFitter function from the lifelines Python module. Significance is tested for the survival of patients with tumors with any RT vs no RT within either the p53 mutant or p53 wildtype cohorts, and within the under 50 and over 50 cohorts, using a log-rank test. In all 4 comparisons, overall survival was significantly worse in patients with any RT.

We also compared survival for any RT vs no RT on an indication-by-indication basis. For many indications, most samples fall into a single category, so we repeated the indication-by-indication analysis comparing ≤ median RT vs > median RT and ≤ median RNA expression vs ≥ median RNA expression.

On an indication-by-indication basis, there was no clear survival trend. Stratifying by median RT calls, three tumor types achieved a significance of p-value < 0.05, two with worse survival in the L1 high group, one with better survival in the L1 high group: esophageal cancer (better survival for L1 high), low grade glioma (worse survival for L1 high) and endometrial cancer (worse survival for L1 high). Stratifying by median RNA expression, 6 tumor types achieved survival associations with p-value < 0.05, four with worse survival in the L1 high group, and two with better survival in the L1 high group: bladder cancer (p value 0.03, worse survival for L1 high), cervical cancer (p=0.02, worse survival for L1 high), clear cell renal cancer (p=0.05, better survival for L1 high), pheochromocytomas and paragangliomas (p=0.03, worse survival for L1 high), rectal adenocarcinoma (p=0.02, better survival for L1 high) and sarcoma (p=0.001, worse survival for L1 high).

Supplementary References

1 Ewing, A. D. a. S., Nathan and Sanchez-Luque, Francisco J and Faivre, Jamila and Brennan, Paul M and Richardson, Sandra R and Cheetham, Seth W and Faulkner, Geoffrey J. Nanopore sequencing enables comprehensive transposable element epigenomic profiling. *Molecular Cell* **80**, 915-928 (2020).

2 Rodriguez-Martin, B. *et al.* Pan-cancer analysis of whole genomes identifies driver rearrangements promoted by LINE-1 retrotransposition. *Nat Genet* **52**, 306-319 (2020). https://doi.org/10.1038/s41588-019-0562-0

3 Ebert, P. *et al.* Haplotype-resolved diverse human genomes and integrated analysis of structural variation. *Science* **372** (2021). https://doi.org/10.1126/science.abf7117

4 Gao, J. *et al.* Integrative analysis of complex cancer genomics and clinical profiles using the cBioPortal. *Sci Signal* **6**, pl1 (2013). https://doi.org/10.1126/scisignal.2004088

5 Brouha, B. *et al.* Hot L1s account for the bulk of retrotransposition in the human population. *Proc Natl Acad Sci U S A* **100**, 5280-5285 (2003). https://doi.org/10.1073/pnas.0831042100

6 Levin, H. L. & Moran, J. V. Dynamic interactions between transposable elements and their hosts. *Nat Rev Genet* **12**, 615-627 (2011). https://doi.org/10.1038/nrg3030

7 Kazazian, H. H., Jr. & Moran, J. V. The impact of L1 retrotransposons on the human genome. *Nat Genet* **19**, 19-24 (1998). https://doi.org/10.1038/ng0598-19

8 Ostertag, E. M. & Kazazian, H. H., Jr. Twin priming: a proposed mechanism for the creation of inversions in L1 retrotransposition. *Genome Res* **11**, 2059-2065 (2001). https://doi.org/10.1101/gr.205701

9 Ostertag, E. M. & Kazazian, H. H., Jr. Biology of mammalian L1 retrotransposons. *Annu Rev Genet* **35**, 501-538 (2001). https://doi.org/10.1146/annurev.genet.35.102401.091032

10 Szak, S. T. *et al.* Molecular archeology of L1 insertions in the human genome. *Genome Biol* **3**, research0052 (2002). https://doi.org/10.1186/gb-2002-3-10-research0052

11 Richardson, S. R. *et al.* The Influence of LINE-1 and SINE Retrotransposons on Mammalian Genomes. *Microbiol Spectr* **3**, MDNA3-0061-2014 (2015). https://doi.org/10.1128/microbiolspec.MDNA3-0061-2014

12 Li, W. & Godzik, A. Cd-hit: a fast program for clustering and comparing large sets of protein or nucleotide sequences. *Bioinformatics* **22**, 1658-1659 (2006). https://doi.org/10.1093/bioinformatics/btl158

13 Fu, L., Niu, B., Zhu, Z., Wu, S. & Li, W. CD-HIT: accelerated for clustering the next-generation sequencing data. *Bioinformatics* **28**, 3150-3152 (2012). https://doi.org/10.1093/bioinformatics/bts565

14 Frith, M. C., Wan, R. & Horton, P. Incorporating sequence quality data into alignment improves DNA read mapping. *Nucleic Acids Res* **38**, e100 (2010). https://doi.org/10.1093/nar/gkq010

15 Hubley, R. *et al.* The Dfam database of repetitive DNA families. *Nucleic Acids Res* **44**, D81-89 (2016). https://doi.org/10.1093/nar/gkv1272

16 Penzkofer, T. *et al.* L1Base 2: more retrotransposition-active LINE-1s, more mammalian genomes. *Nucleic Acids Res* **45**, D68-D73 (2017). https://doi.org/10.1093/nar/gkw925

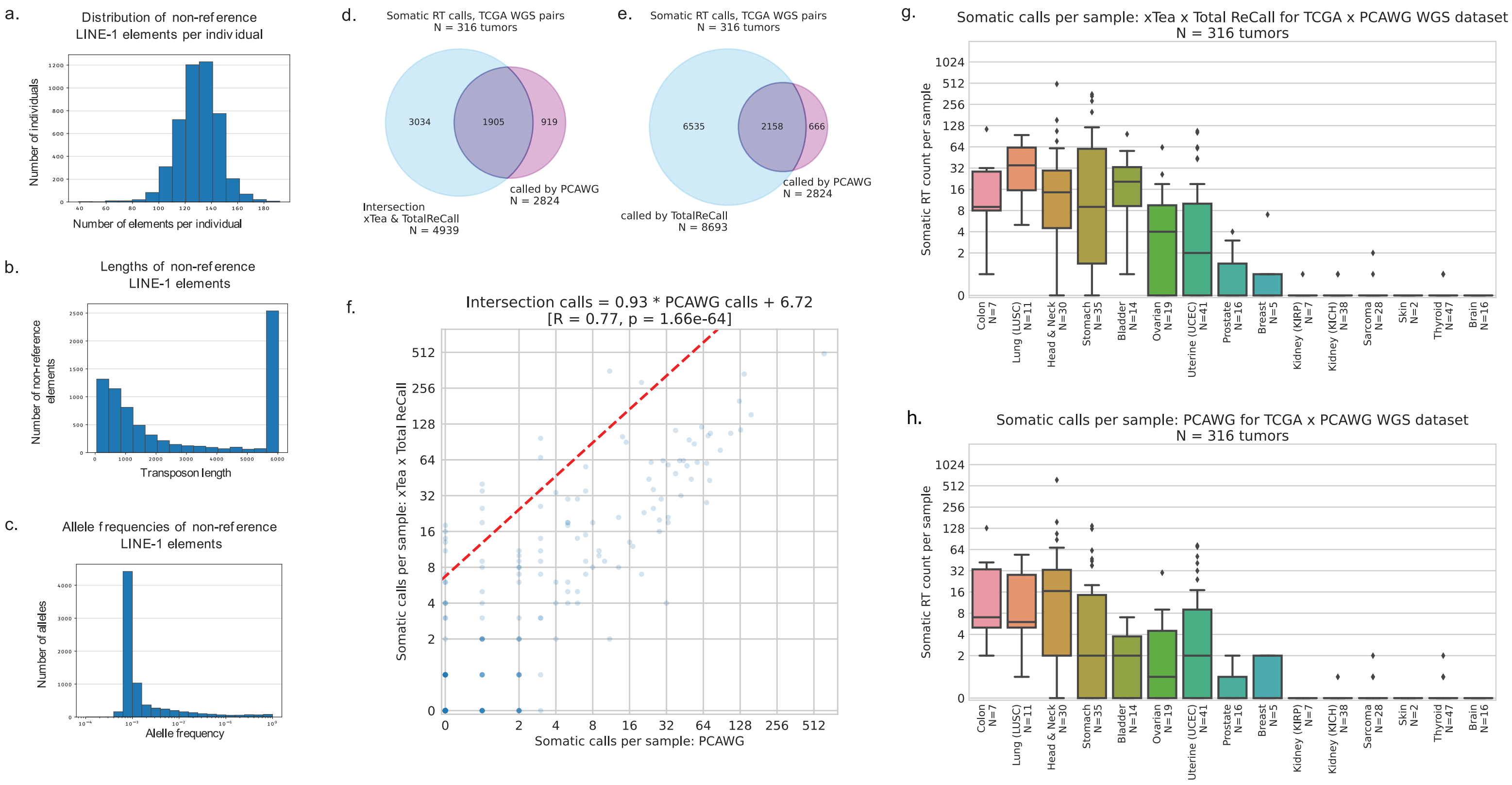

Supplementary Figure 1 | Properties of non-reference LINE-1 elements and comparison of RT call set from this paper to the PCAWG call set for all shared tumor samples. a) Number of non-reference LINE-1 elements per individual across all samples for which there was paired WGS data. b) Distribution of lengths of non-reference LINE-1 elements, across all non-reference LINE-1 elements detected. c) Allele frequencies of all non-reference LINE-1 elements as calculated for the set of individuals for which paired tumor and normal WGS samples were available. d) Venn diagram of total calls throughout dataset from our call set (the intersection of TotalReCall with xTea calls) and the PCAWG call set. e) Venn diagram of total calls throughout the dataset from TotalReCall alone and PCAWG. f) Correlation between sample-level count of calls from our call set (y-axis) vs. PCAWG (x-axis). R=0.77, p < 10-10, Pearson correlation. Red line shows linear regression fit, ordinary least squares. g) Somatic RT count per sample from our call set, grouped by tumor type. h) Somatic RT count per sample from the PCAWG call set, grouped by tumor type. g-h) Center line indicates median. Box indicates interquartile range. Points more than 1.5 x IQR away from the box are shown as individual outliers. Tumor types are sorted as in Figure 2. (a-c) N = 4667 tumor-normal pairs. (d-g) N = 316 tumors.

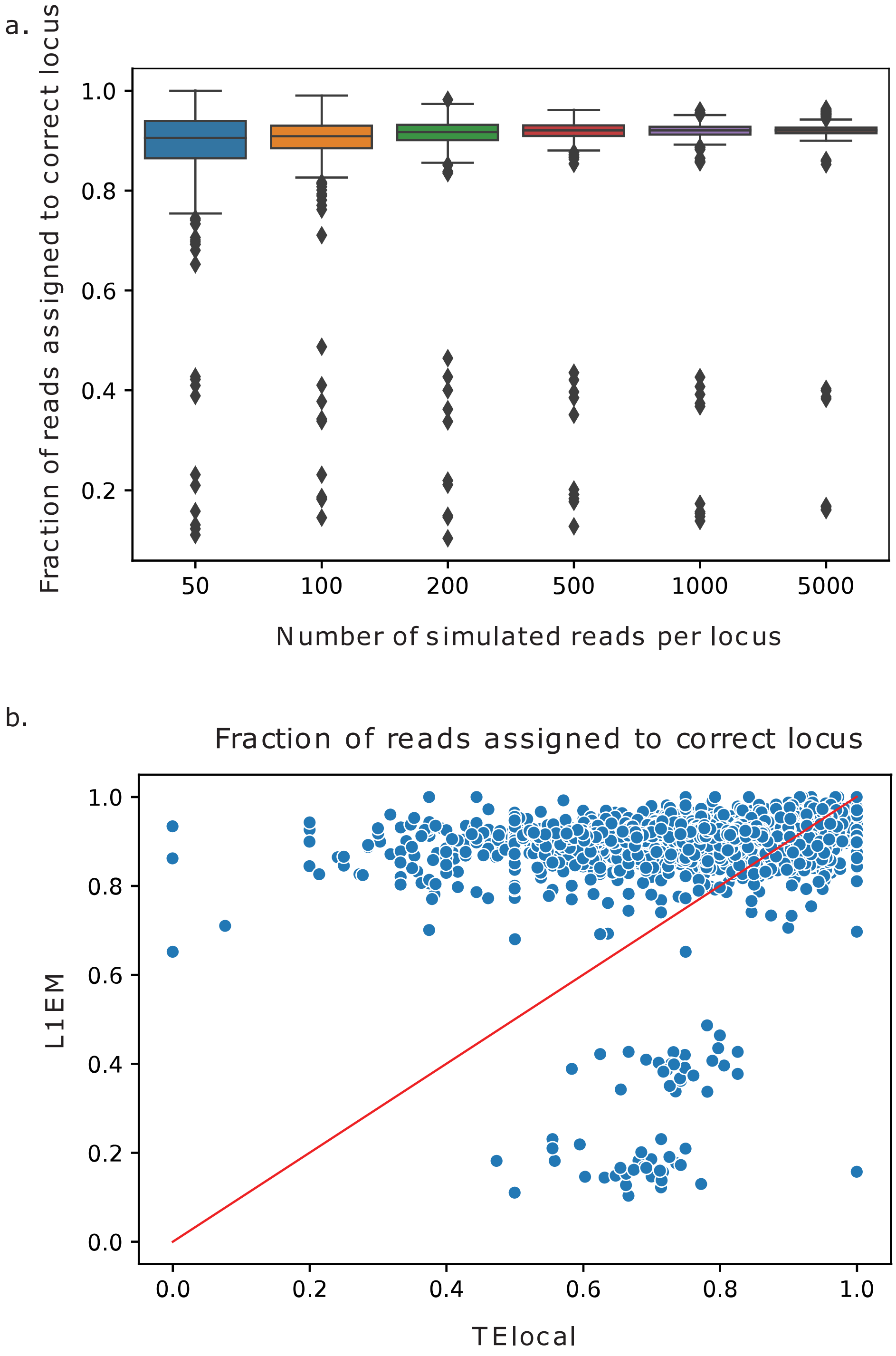

Supplementary Figure 2 | in silico evaluation of locus-level quantification accuracy by L1EM. a) Fraction of reads correctly assigned to reference locus by L1EM across all full-length intact L1 loci, with simulations ranging from 50 to 5000 reads per locus, indicating that L1EM typically assigns ~90% of reads accurately at the locus level. b) Comparison of the L1EM results in a) to the results obtained using TElocal for the same set of simulated data. The red line indicates x=y.

a.

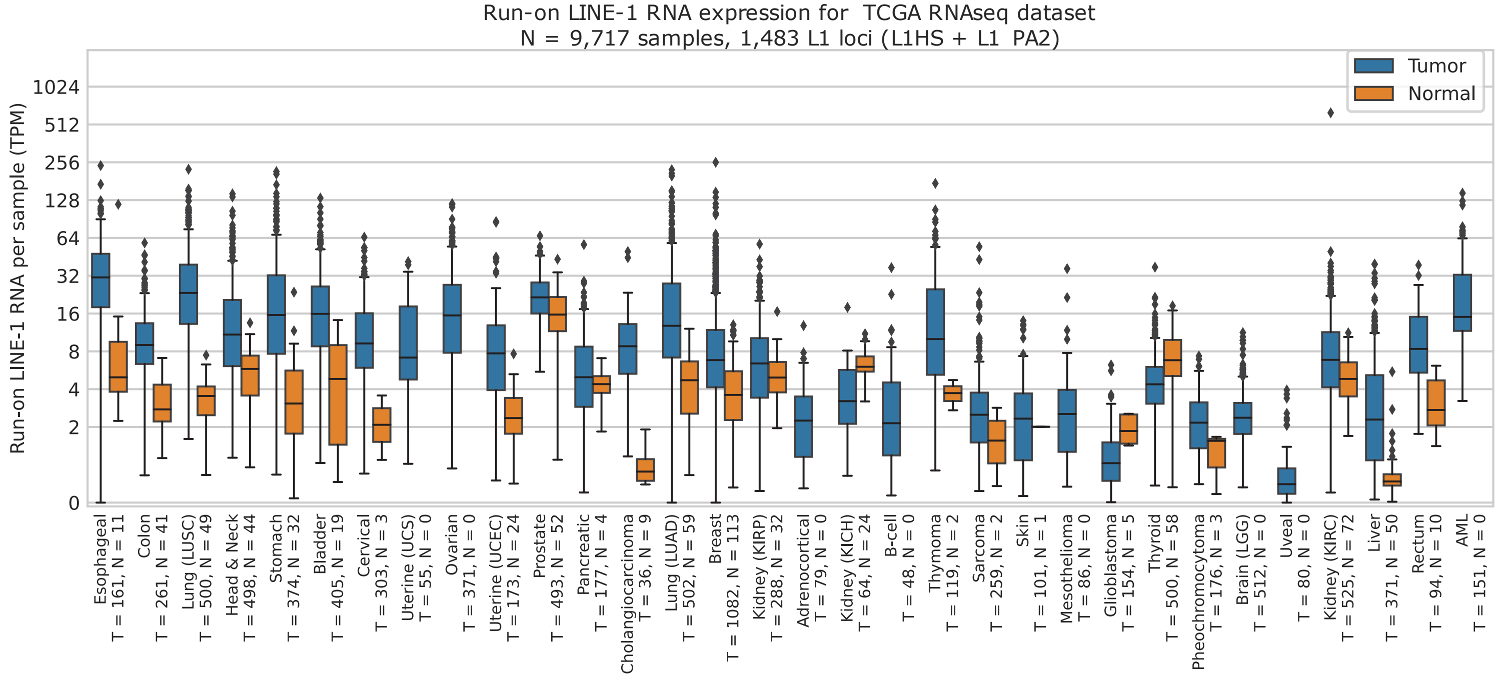

b.

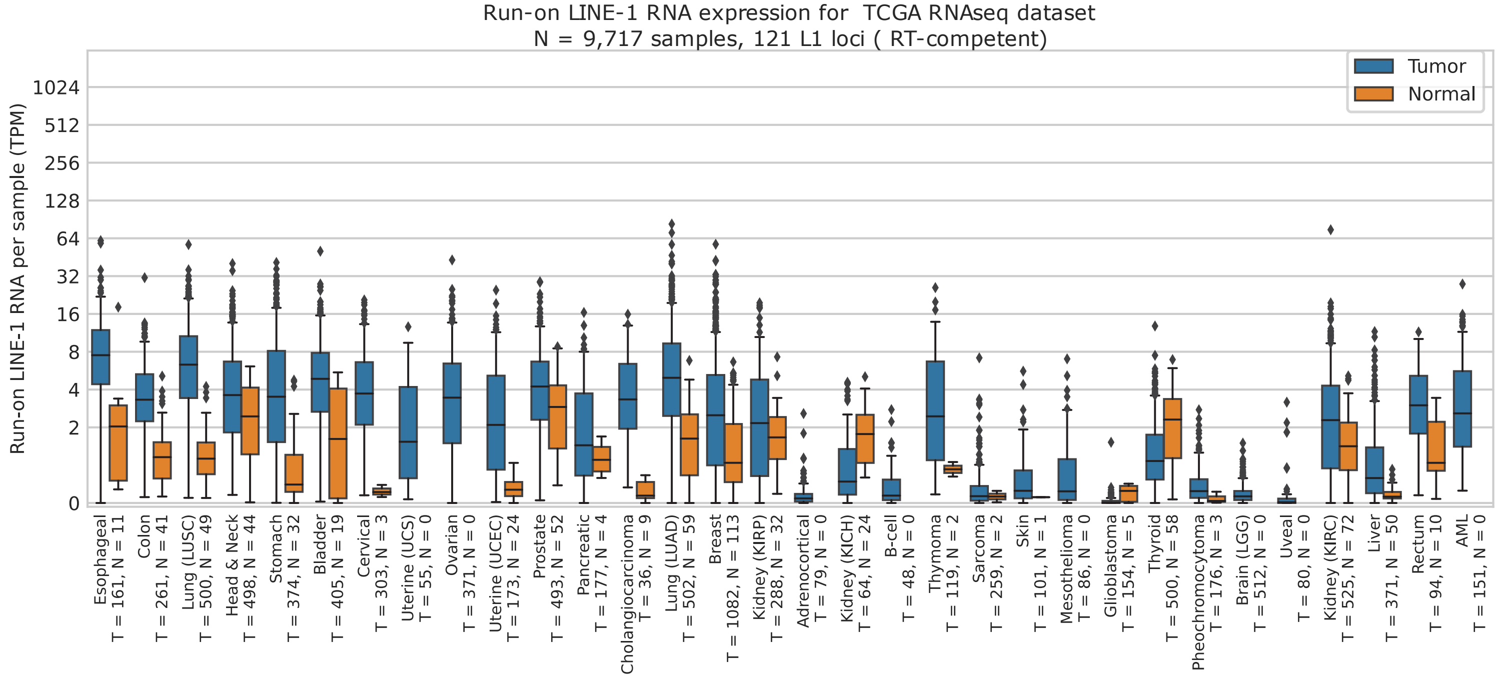

Supplementary Figure 3 | Estimated expression of run-on L1 RNA in each sample grouped by tumor type. Quantified by L1EM. Total N = 9,717 samples; 8,998 tumor samples and 719 normal samples. Tumor types are sorted as in Figure 2, with the addition of rectal adenocarcinoma and AML. Blue, tumor samples. Orange, normal samples. a) L1 RNA expression per sample is aggregated across all 1,483 L1HS and L1PA2 loci in L1EM. b) L1 RNA expression per sample is aggregated across 121 loci for which there is evidence of in vitro transductions, whether from this study or annotated in Ebert et al[3].

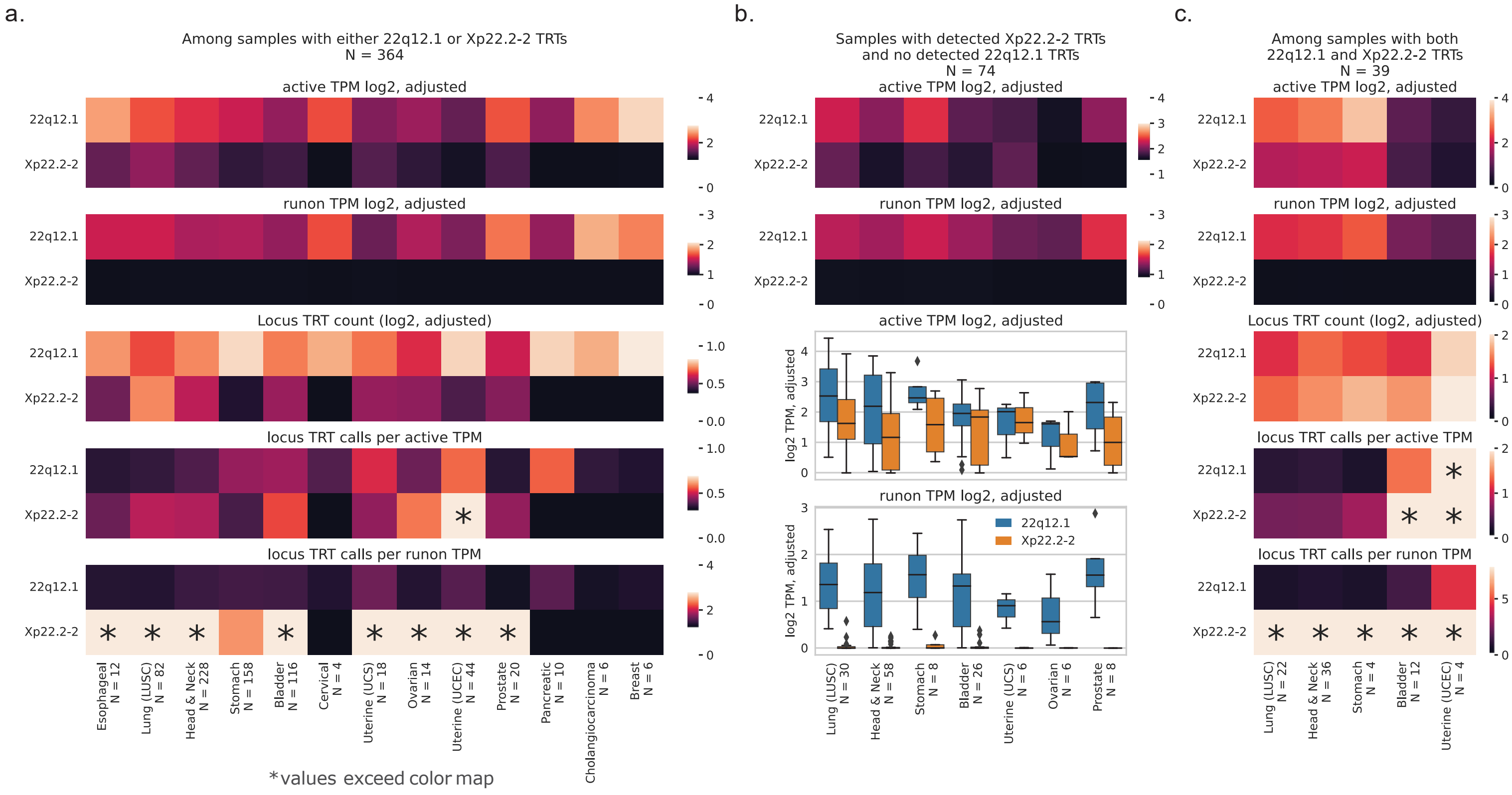

Supplementary Figure 4 | Comparing Xp22.2-2 and 22q12.1 RNA and transductions within subset of tumor samples with transductions. a) RNA expression and transduction counts for L1 elements 22q12.1 and Xp22.2-2 across tumor types, within the subset of samples with at least one observed transduction from either element. N = 364 tumors. Heatmaps from top to bottom: mean active ("only" + "run-on" transcripts) RNA expression from each element per sample within each tumor type, log2 TPM adjusted values. Mean run-on RNA expression from each element per sample within each tumor type, log2 TPM adjusted values. Mean identified transductions per sample from each locus within each tumor type, log2 adjusted count values. Ratio of mean identified transductions per sample (counts) / mean active RNA expression per sample (TPM) from each locus within each tumor type. Ratio of mean identified transductions per sample (counts) / mean run-on RNA expression per sample (TPM) from each locus within each tumor type. Although transductions are clearly possible within all 364 tumors, and 22q12.1 is expressed notably higher in both active and run-on RNA, Xp22.2-2 makes proportionately more transductions per unit RNA. b) RNA expression for both elements across tumor types, within the subset of samples where transductions have been observed for Xp22.2-2 but not 22q12.1. N = 74 tumors. Above, heatmaps showing mean active RNA expression per sample and mean run-on RNA expression per sample for each locus within each tumor type, log2 TPM adjusted values. Below, box plots showing the distribution of active RNA expression and run-on RNA expression for all samples within each tumor type, log2 TPM adjusted values. Blue, expression of 22q12.1. Orange, expression of Xp22.2-2. Center lines indicate median, box indicates interquartile range, and points more than 1.5 x IQR away from the IQR box are shown as individual outliers. 22q12.1 is higher expressed than Xp22.2-2 within these samples, both in active RNA and run-on, despite not creating any transductions. c) RNA expression and transduction counts for both elements across tumor types, within the subset of samples with at least one observed transduction from both elements, and tumor types with at least 3 of such samples. N = 39 tumors. Heatmap sections as in (a). All 39 tumors must have at least one functional allele of both elements, and 22q12.1 is more highly expressed, yet Xp22.2-2 makes slightly more transductions total, and notably more transductions per unit RNA.

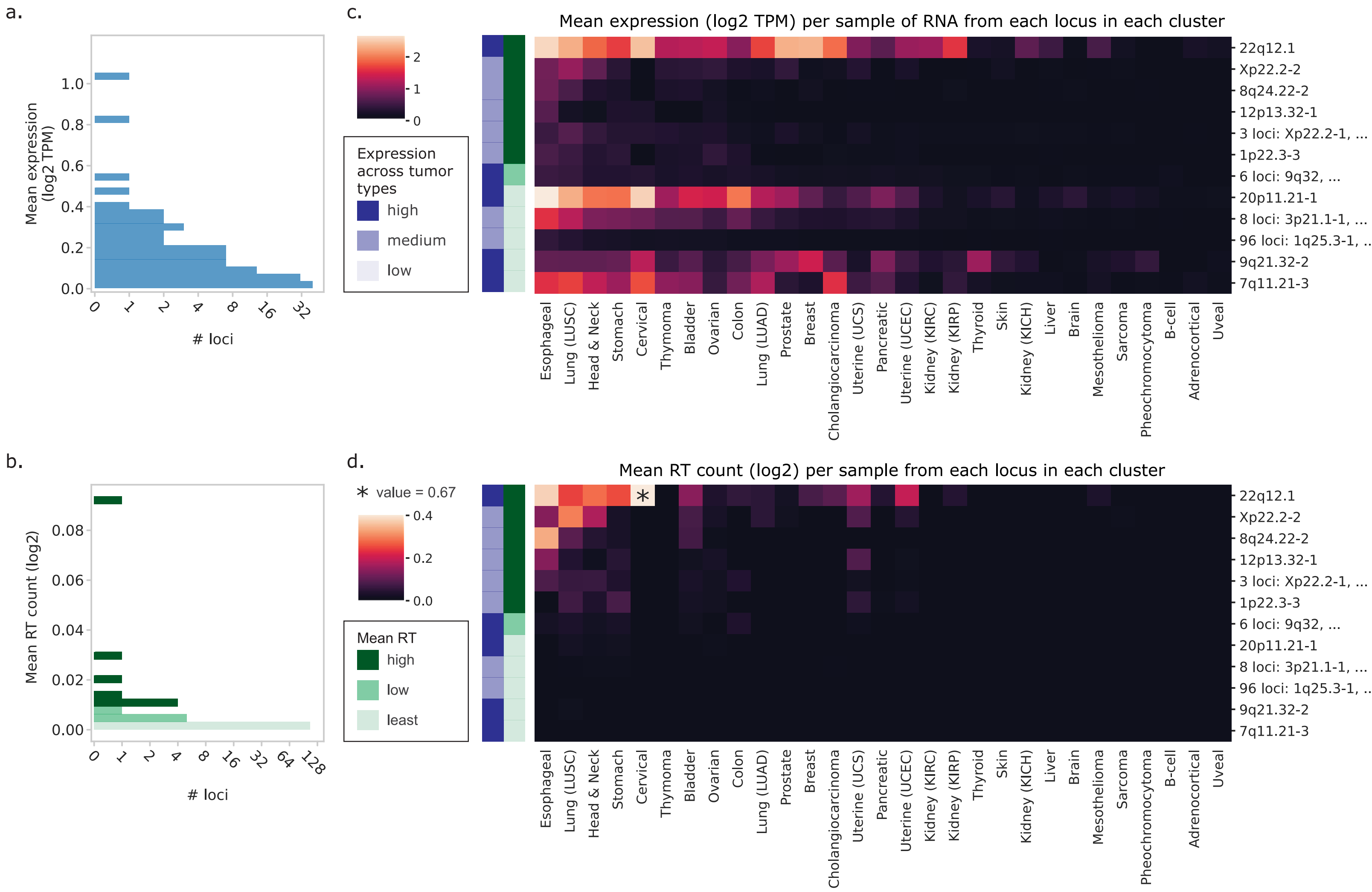

Supplementary Figure 5 | Landscape of L1 locus activity across tumor types, for 121 L1 loci with observed transductions in vitro. a) Histogram of per-sample mean L1 RNA expression (log2 TPM) for all 121 L1HS and L1PA2 loci. Mean expression per sample is weighted by the inverse of samples with the same tumor type, such that each tumor type contributes equally to the locus mean. b) Histogram of per-sample mean L1 RT (log2 count) (based on identified transductions) for the same 121 L1HS and L1PA2 loci. Mean RT count per sample is weighted by the inverse of samples with the same tumor type, such that each tumor type contributes equally to the locus mean. c) Heatmap of mean L1 RNA expression (log2 TPM) of each locus within a given cluster (rows) across tumor types (columns). d) Heatmap of mean log2 L1 RT count (based on identified transductions) of each locus within a given cluster (rows) across tumor types (columns). c-d) The 121 L1HS and L1PA2 loci have been clustered based on similar expression and RT count profiles across tumor types, resulting in 12 clusters. The same clusters are used in c) and d), with the same ordering for columns and rows. Clusters are named based on the locus in each cluster with the highest mean RNA expression. To generate each heatmap value, a mean for each locus within each tumor type is first calculated, and then the mean of means for all loci within a cluster. Clusters are sorted from top to bottom by highest to lowest mean RT value. Rows are sorted left to right by highest to lowest total L1 RNA expression (summed across all 121 loci) per sample. To the left of each heatmap, the row colors annotate each cluster categorically based on RNA and RT. The left column (dark, medium, and light blue) indicates the distribution of RNA expression of each cluster across tumor types. “High”, indicates clusters expressed in >15 tumor types, with expression defined as having mean expression >= 0.1 TPM. “Medium” indicates clusters expressed in between 5 and 15 tumor types. “Low” indicates clusters expressed in fewer than 5 tumor types. The right column (dark, medium, and light green) indicates the mean RT count of all loci within the cluster, categorized into “high” (dark green), “low” (medium green), and “least” (light green) based on the histogram in b).

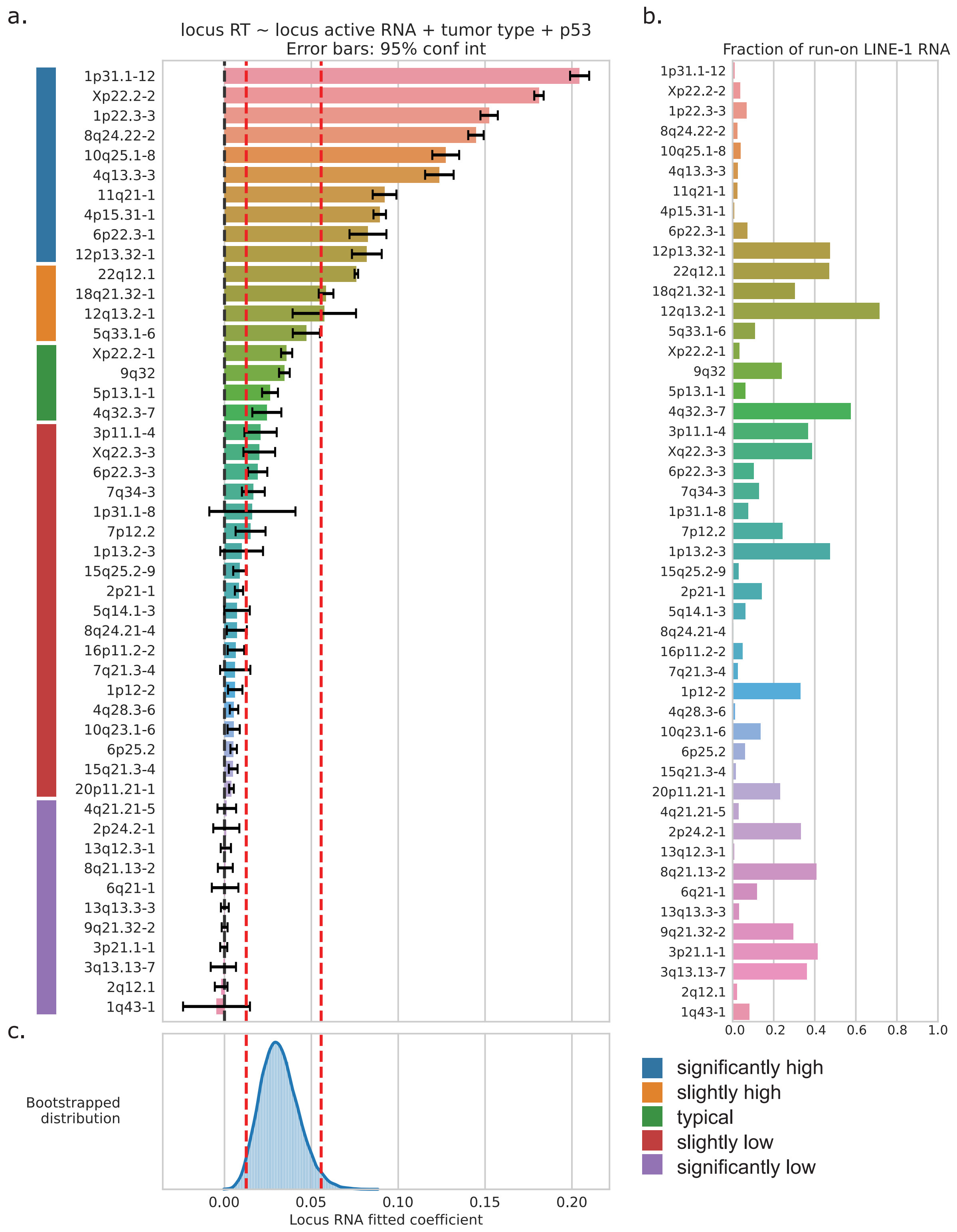

Supplementary Figure 6 | Fitting regression models to represent locus efficiency. Locus RT (log2, adjusted) counts regressed as a function of locus RNA (log2, adjusted TPM) + tumor type + p53 mutation, fitted using ordinary least squares. Models were fitted based on 3,820 tumor samples for which we have WGS, RNA, and p53 data. a) Coefficients assigned to locus RNA for 48 loci with transductions in at least 2 individuals in this study. Bars represent fitted coefficient. Error bars represent 95% confidence intervals. Colored bars to the left indicate which category each locus was assigned to in Figure 6a. b) Fraction of all RNA expressed from each locus that were run-on transcripts. Although run-on transcripts will be the source of transductions, differing relative abundances do not explain the differences in locus efficiency found in (a). c) Histogram of coefficients assigned to background loci generated by randomly resampled permutations of these 48 loci, N = 1,000 permutations, as seen in Figure 6a. Red lines indicate 95% confidence interval of background locus coefficients.

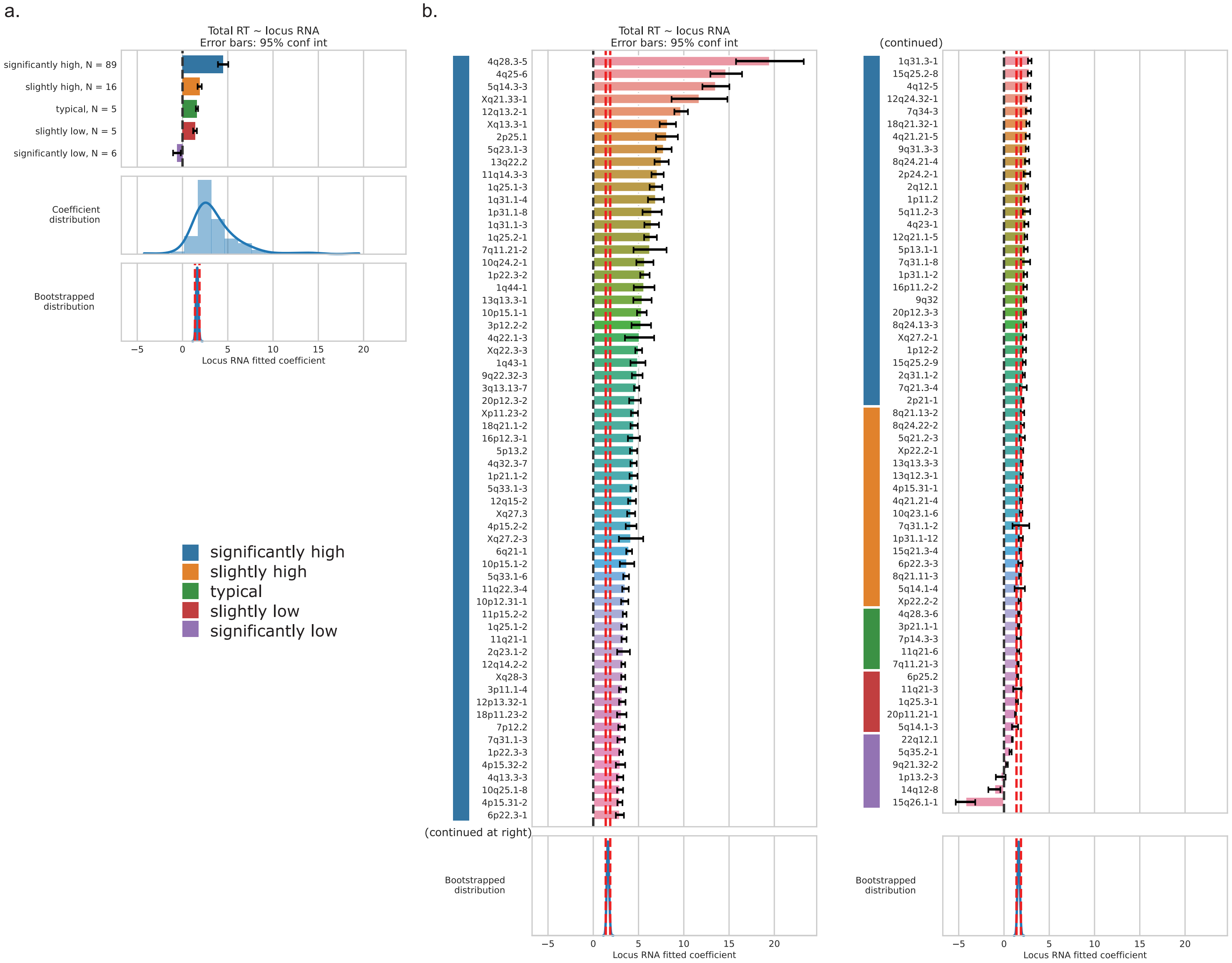

Supplementary Figure 7 | Fitting regression models to correlate locus expression with total RT burden. Total RT (log2, adjusted) counts regressed as a function of locus RNA (log2, adjusted TPM), fitted using ordinary least squares. Models were fitted for 121 loci with evidence of generating transductions, whether observed in this or a previous[26] study. Background distribution was generated by randomly resampled permutations of these 121 loci, N = 1,000 permutations. Models were fitted based on 3,879 tumor samples for which we have WGS and RNA data. a) Fitted coefficients assigned to locus RNA. Top, coefficients for 121 loci are grouped into five categories based on where they fall relative to the bootstrapped background distribution. Each bar represents the mean coefficient assigned to the loci within that category, and the error bars represent the mean of the lower and upper bounds of 95% confidence intervals for those coefficients. “Significantly high” or “Significantly low” categories include loci that were assigned coefficient values that gave a one-sided multiple hypothesis-corrected p-value ≤ 0.05 when compared to the background distribution. “Slightly high” or “Slightly low” categories include loci that were assigned coefficient values falling outside the interquartile range of the background distribution. “Typical” category includes loci with coefficient values within the interquartile range of the background distribution. Middle, histogram of coefficients assigned to these 121 loci. Variance = 1.71, p < 0.001 based on variances of background permutations. Bottom, histogram of coefficients assigned to background loci generated by randomly resampled permutations of these 121 loci, N = 1,000 permutations. Red lines indicate 95% confidence interval of background locus coefficients. b) Top, coefficients assigned to locus RNA for 121 loci. First 61 loci are shown to the left, and remaining 60 loci continue to the right. Bars represent fitted coefficients. Error bars represent 95% confidence intervals. Colored bars to the left of each locus name indicate which category each locus was assigned to in (a). Bottom, histogram of coefficients assigned to background loci, as shown at the bottom of (a). Red lines indicate 95% confidence interval of background locus coefficients.

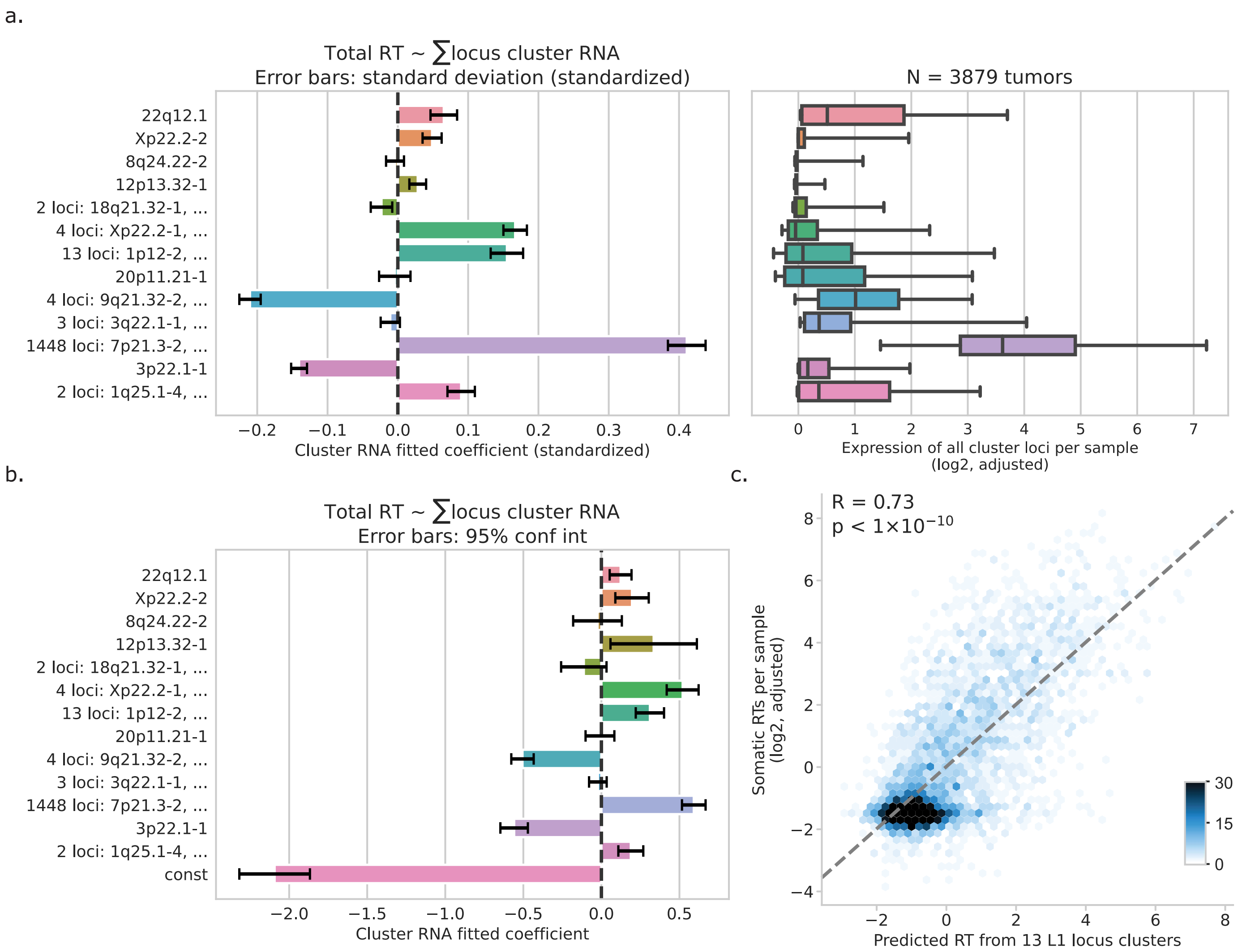

Supplementary Figure 8 | Fitting a single regression model of total RT burden as a function of the expression of RNA from each cluster of loci. 13 clusters of 1,483 L1HS and L1PA2 loci defined in Figure 4 based on similar profiles of RNA expression and RT counts across tumor types. Total RT (log2, adjusted) counts regressed as a function of the sum of cluster RNA (log2, adjusted TPM, summed across loci within each cluster), fitted using ordinary least squares. Model was fitted based on 3,879 tumor samples for which we have WGS and RNA data. a) Left, fitted coefficients assigned to each cluster, standardized by the standard deviation of total RT / standard deviation of RNA expression from that cluster. Bars represent fitted coefficient. Error bars represent standardized standard deviation. Right, box plot representing the distribution of total RNA expression (log2, adjusted) for RNA from loci belonging to the cluster across 3,879 tumor samples. Center line indicates mean. Box indicates interquartile range. Whiskers extend to 95% confidence interval; individual outliers outside of this interval not shown for clarity. b) Fitted coefficients assigned to each cluster, unstandardized. Bars represent fitted coefficients. Error bars represent 95% confidence intervals. c) Actual somatic RT burden per sample (y-axis) vs. prediction based on model fit (x-axis). N = 3,879 tumor samples, R = 0.73, $p < 10^{-10}$, Pearson correlation (using the exact distribution, as calculated by the scipy.stats.pearsonr function in python). Dashed line represents y = x.

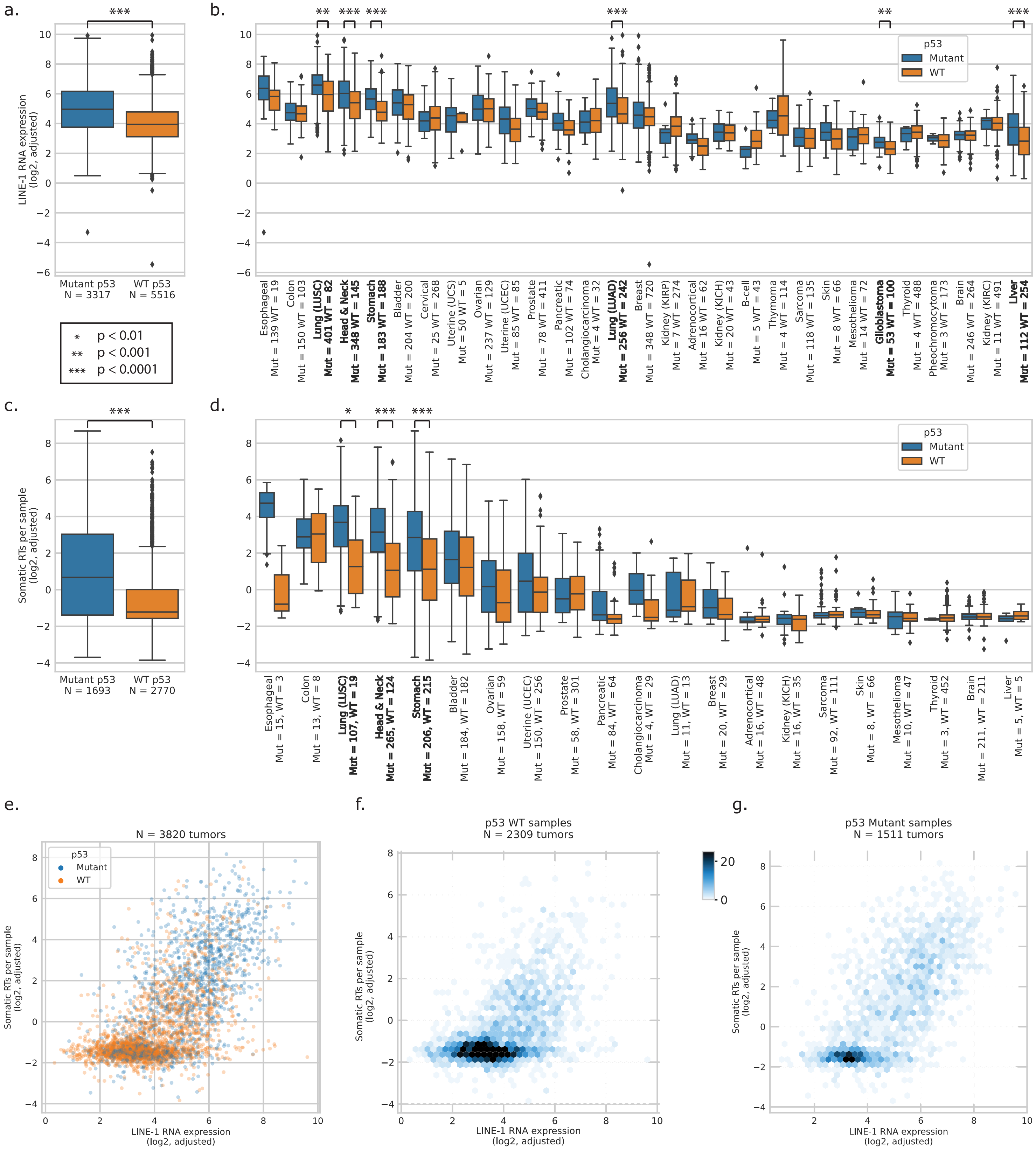

Supplementary Figure 9 | Stratifying L1 RNA and RT by p53 mutation.

Supplementary Figure 9 | Stratifying L1 RNA and RT by p53 mutation. a) Box plots representing L1 RNA expression (log2 TPM, adjusted) within all samples with mutated p53 (N = 3,317 tumors) or WT p53 (N = 5,516 tumors). Total N = 8,833 tumors for which we have both RNA and p53 data. $p < 10^{-10}$, two-sided Mann-Whitney U test (effect size = 0.37, N1 = 3317, N2 = 5516). b) Box plots representing L1 RNA expression (log2 TPM, adjusted, y-axis shared with (a)) stratified by tumor type and p53 mutation. Tumor types with a significant difference between RNA expression in mutant and WT p53 samples highlighted in bold, as calculated by one-sided Mann-Whitney U test. Bonferroni-adjusted p-values and effect sizes for the starred tumor types are as follows. LUSC: 5.11×10^{-4}, 0.29; Head & Neck: 8.6×10^{-6}, 0.29; Stomach: 2.24 x 10-11, 0.43; LUAD: 5.77×10^{-5}, 0.24; Glioblastoma: 4.34×10^{-4}, 0.41; Liver: 7.79×10^{-6}, 0.33. c) Box plots representing L1 RT burden (log2 count, adjusted) within all samples with mutated p53 (N = 1693 tumors) or WT p53 (N = 2,770 tumors). Total N = 4,463 tumors for which we have both WGS and p53 data. p < 10-10, two-sided Mann-Whitney U test (effect size = 0.35, N1 = 1693, N2 = 2770). d) Box plots representing L1 RT burden (log2 count, adjusted, y-axis shared with (c)) stratified by tumor type and p53 mutation. Tumor types with a significant difference between RT burden in mutant and WT p53 samples highlighted in bold, as calculated by one-sided Mann-Whitney U test. Bonferroni-adjusted p-values and effect sizes for the starred tumor types are as follows. LUSC: 7.87×10^{-3}, 0.5; Head & Neck: 9.45×10^{-15}, 0.51; Stomach: 6.47×10^{-9}, 0.35. a-d) Asterisks above indicate the significance level of multiple hypothesis-corrected p-value from a two-sided Mann-Whitney U test (* $p < 0.01$, ** $p < 0.001$, *** $p < 0.0001$). Center lines indicate mean. Boxes indicate interquartile range. Points more than 1.5 x IQR away from the IQR box are shown as individual outliers. Blue boxes, tumors with mutant p53. Orange boxes, tumors with wildtype p53. e) Overall correlation between L1 RT (log2 count, adjusted) and L1 RNA (log2 TPM, adjusted), colored by p53 mutation status. N = 3,820 tumors with WGS, RNA, and p53 category data. Blue dots, p53 mutant tumors. Orange dots, p53 WT tumors. R = 0.65, $p < 10^{-10}$, Pearson correlation (using the exact distribution, as calculated by the scipy.stats.pearsonr function in python). f) Correlation between L1 RT and L1 RNA within p53 WT tumors only, N = 2,309. R = 0.52, $p < 10^{-10}$, Pearson correlation (exact distribution). g) Correlation between L1 RT and L1 RNA within p53 mutant tumors only, N = 1,511. R = 0.69, $p < 10^{-10}$, Pearson correlation (exact distribution). a-g) p53 mutant or WT classification based on annotations from cBioPortal.

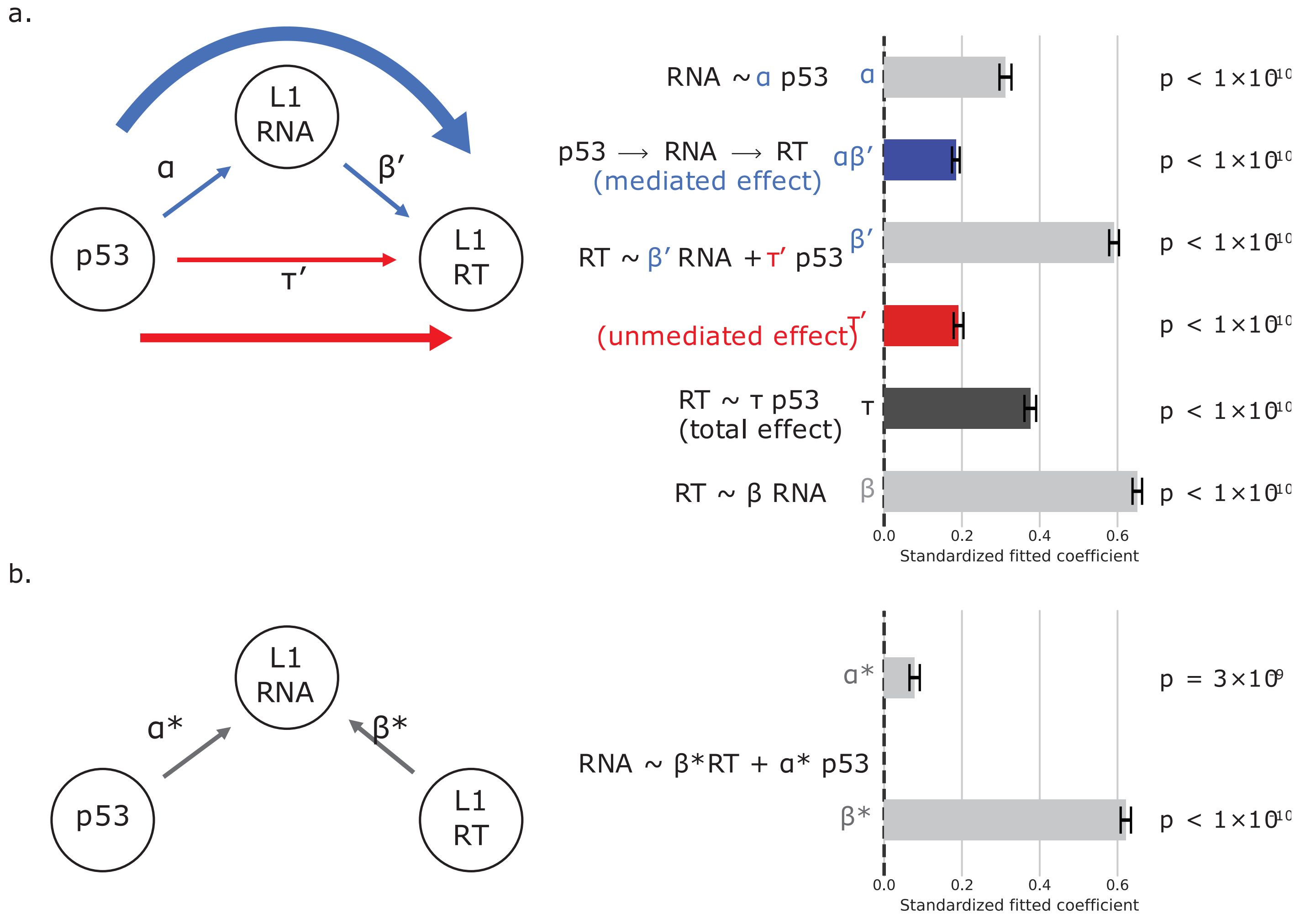

Supplementary Figure 10 | Breakdown of linear regression fitted coefficients from p53 regulatory mediation model. a) Standardized fitted values for each coefficient within the mediation model and corresponding likelihood. Each coefficient is calculated based on the linear regression model shown to the left. N = 3,793 tumor samples for all models. Error bars indicate standardized standard errors. Dark gray bar represents the total influence of p53 on L1 RT. Red bar represents the unmediated pathway effect. Blue bar represents the mediated pathway effect. Light gray bars represent other coefficient fits of regressions with these variables. b) Standardized fitted values for the reversed model, fitting L1 RNA expression (log2 TPM, adjusted) as a function of L1 RT (log2 count, adjusted) and p53. The significance of α* confirms that p53 has a significant effect on L1 RNA even when controlling for L1 RT burden.

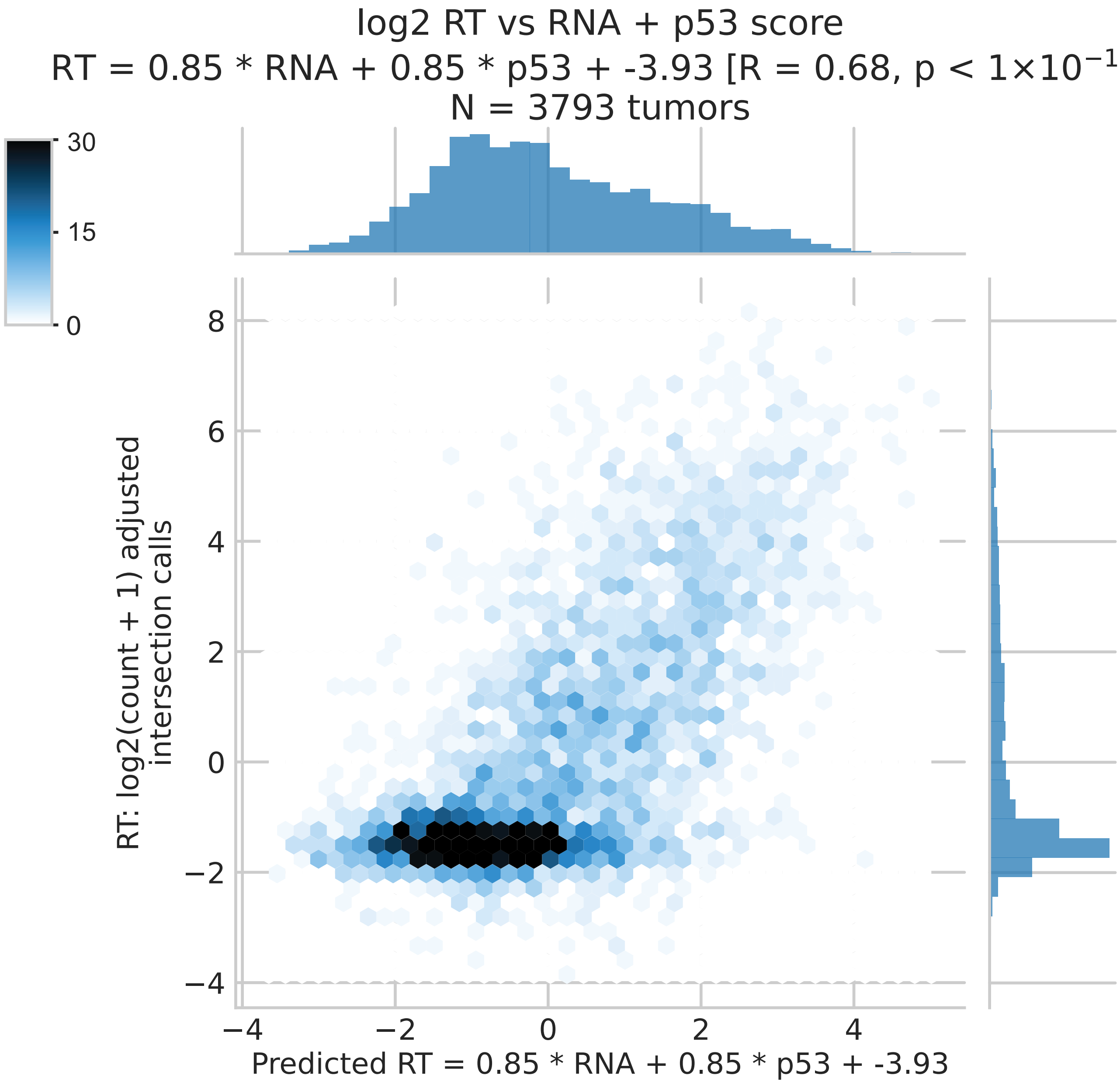

Supplementary Figure 11 | Correlation between L1 RT burden and predicted RT burden based on linear regression of L1 RNA and p53. N = 3,793 tumors. L1 RT expressed as log2 count, adjusted. L1 RNA expressed as log2 TPM, adjusted. p53 expressed as binary value indicating mutated or not mutated. R = 0.68, $p < 10^{-10}$, Pearson correlation.

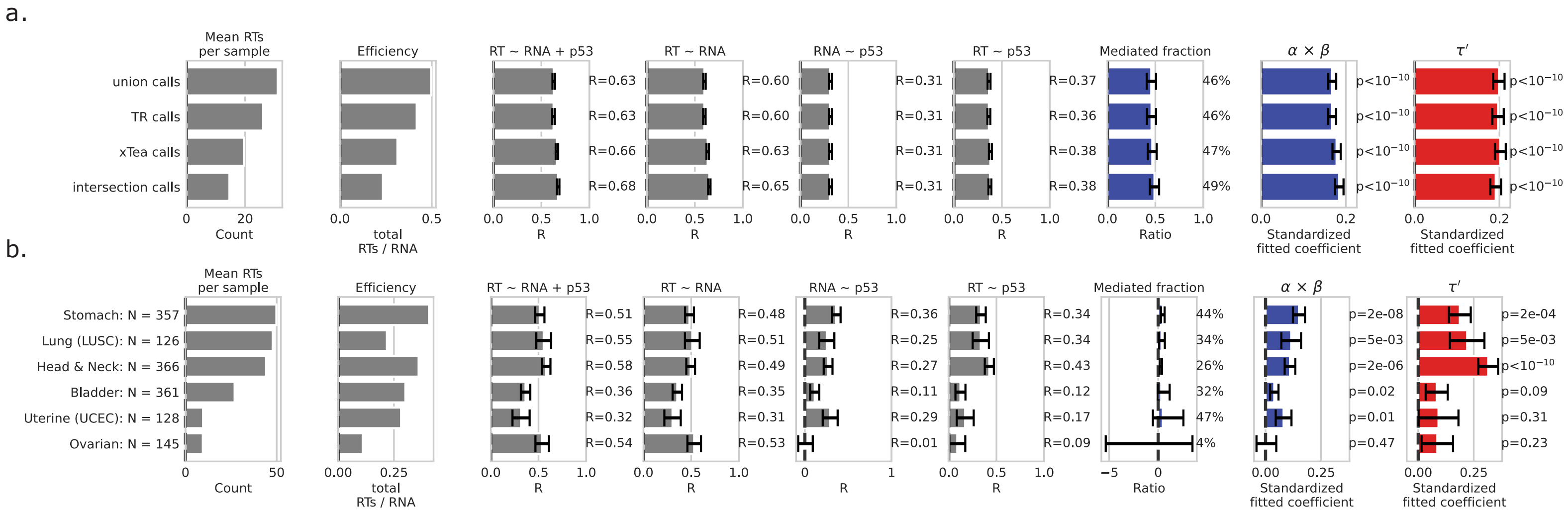

Supplementary Figure 12 | Results of mediation model fits across a range of definitions of variables or stratifications. Columns of bar plots from left to right: Mean RTs per sample within each model's dataset. Overall "efficiency" within each model, expressed as the ratio of total RTs (count) to total RNA (TPM). Correlation coefficient of RT ~ RNA + p53 regression, with error bars for 95% confidence interval. Correlation coefficient of RNA ~ p53 regression, with error bars for 95% confidence interval. Correlation coefficient of RT ~ p53 regression, with error bars for 95% confidence interval. Fraction of total RT ~ p53 effect through the mediated pathway, with error bars for 95% confidence interval. Standardized fitted coefficient for the mediated pathway, with error bars for standardized standard error. Standardized fitted coefficient for the unmediated pathway, with error bars for standardized standard error. a) Mediation models using 4 call sets to define RT burden (the union of calls from TotalReCall or xTea, all calls from TotalReCall, all calls from xTea, and the intersection of calls identified by both callers). In all cases, L1 RT is represented by log2 count, adjusted. L1 RNA is represented by the total expression of 1,483 loci, log2 TPM, adjusted. p53 is represented by a binary indicator of mutated vs. not mutated as annotated by cBioPortal. The results of the model are consistent regardless of which RT call set is used. N = 3,793 for all models. b) Mediation models for tumor samples within a single tumor type. Tumor types with at least 20 tumor samples, non-zero variance in all 3 variables, and a median of at least 1 RT per sample are included. L1 RT is represented by the intersection call set, log2 count adjusted. L1 RNA is represented by the total expression of 1,483 loci, log2 TPM, adjusted. p53 is represented by a binary indicator of mutated vs. not mutated as annotated by cBioPortal.

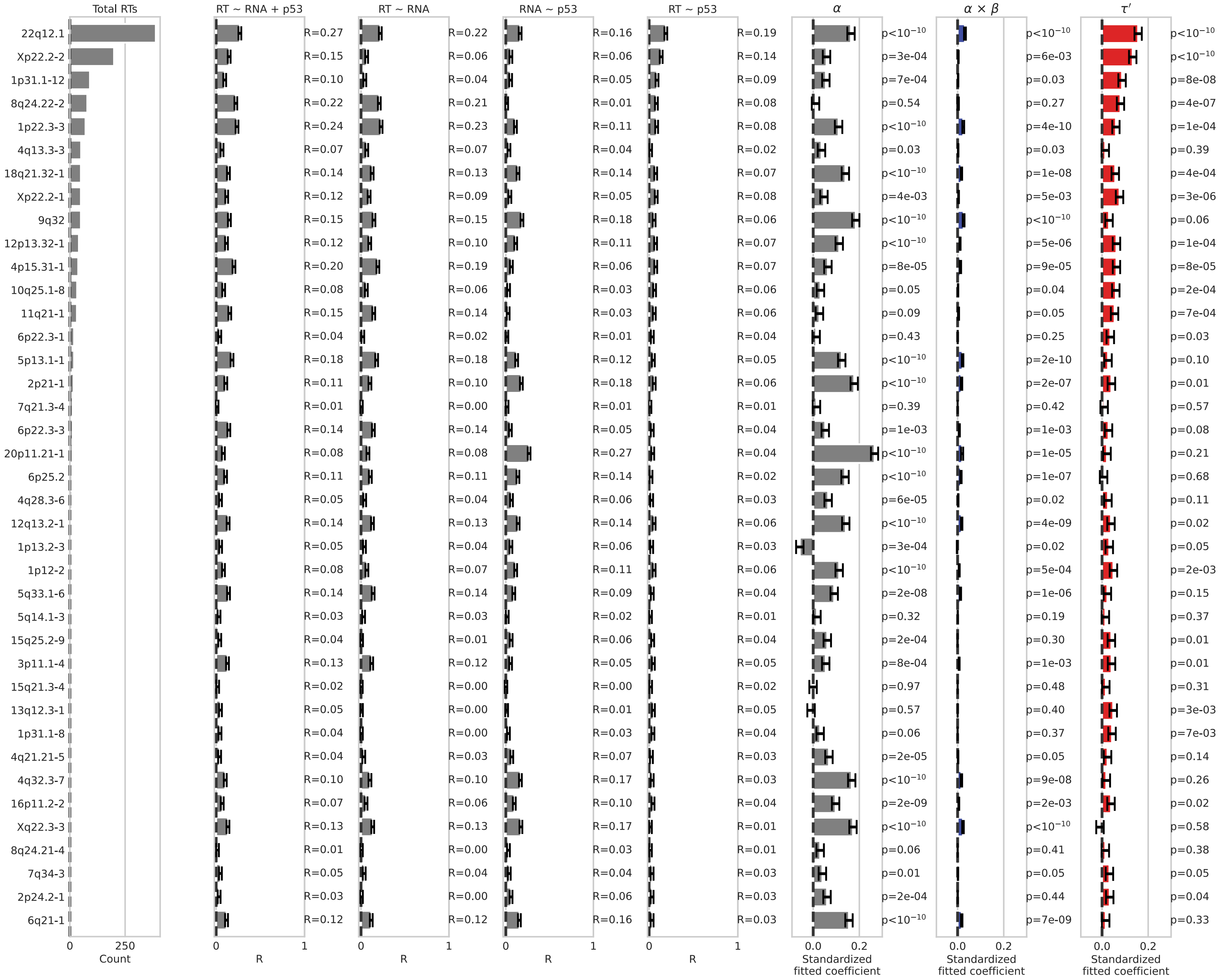

Supplementary Figure 13 | Results of mediation model fits for L1 RT and RNA per locus, using binary mutated value for p53. Columns of bar plots from left to right: Total RTs from that locus throughout the dataset. Correlation coefficient of RT ~ RNA + p53 regression, with error bars for 95% confidence interval. Correlation coefficient of RT ~ RNA regression, with error bars for 95% confidence interval. Correlation coefficient of RNA ~ p53 regression, with error bars for 95% confidence interval. Correlation coefficient of RT ~ p53 regression, with error bars for 95% confidence interval. Standardized fitted coefficient for p53 regulation of L1 RNA, with error bars for standardized standard error. Standardized fitted coefficient for the mediated pathway of p53 regulation of L1 RT, with error bars for standardized standard error. Standardized fitted coefficient for the unmediated pathway of p53 regulation of L1 RT, with error bars for standardized standard error. N = 3,793 tumors for all models.

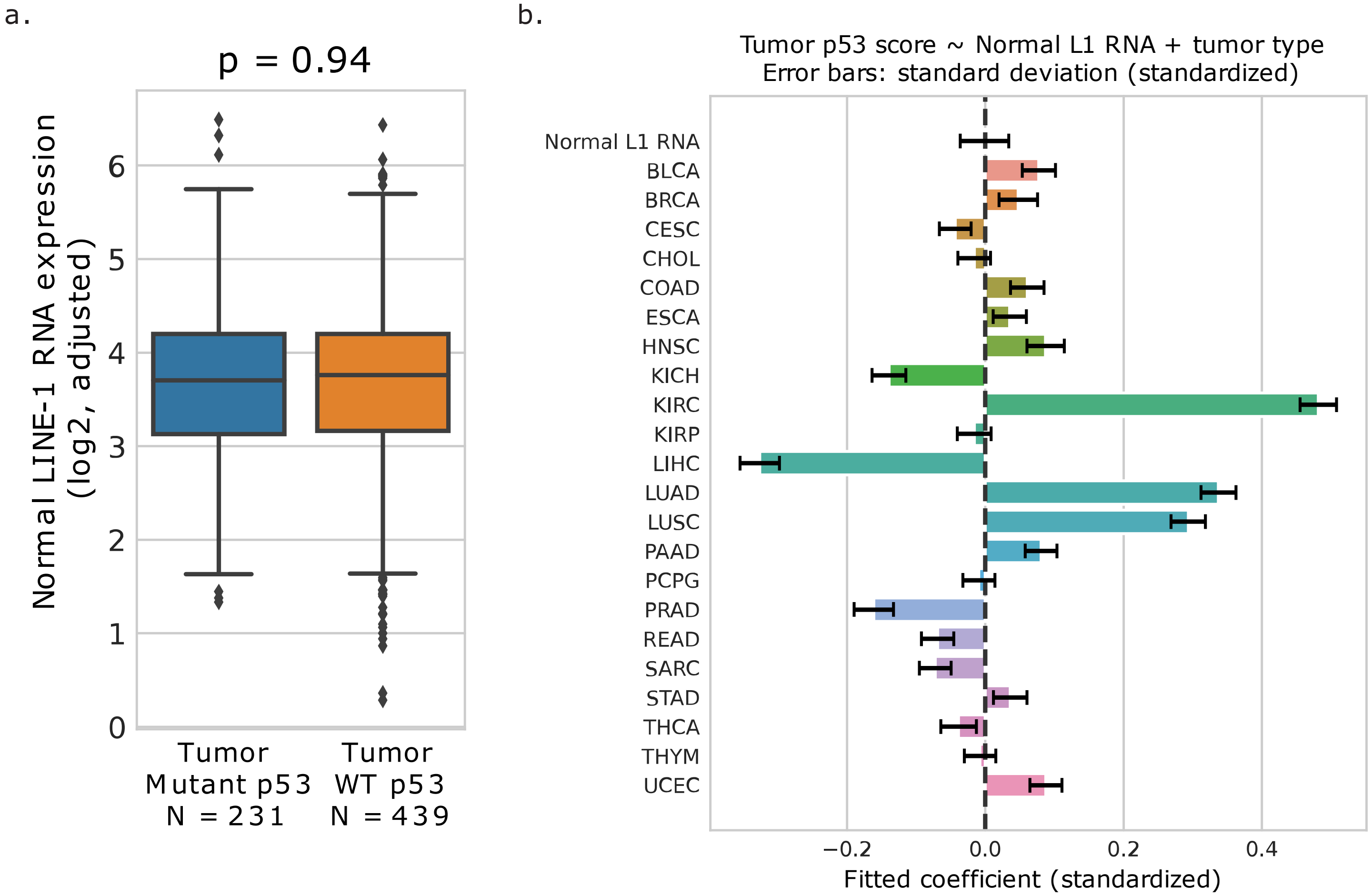

Supplementary Figure 14 | Evaluating normal tissue L1 RNA as a potential selective precursor to tumor p53 mutation. No significant relationship found. a) Box plots comparing the L1 RNA expression (log2 TPM, adjusted) in 670 normal samples, stratified by whether the corresponding tumor samples have p53 mutations, as annotated by cBioPortal. p = 0.94, two-sided Mann-Whitney U test, effect size = -0.003, N1 = 231, N2 = 439. Center line indicates median. Boxes indicate interquartile range. Points more than 1.5 x IQR away from the box are shown as individual outliers. b) Standardized coefficients resulting from using ordinary least squares to fit a linear regression of binary tumor p53 mutation as a function of paired normal L1 RNA (log2 TPM, adjusted) + tumor type. Although many tumor types were either positively or negatively predictive of p53 mutation, normal L1 RNA was assigned an insignificant coefficient near 0. Bars represent the standardized coefficient. Error bars represent the standardized standard error of the coefficient estimate. N = 670 patients with tumor p53 annotation and normal RNA data.

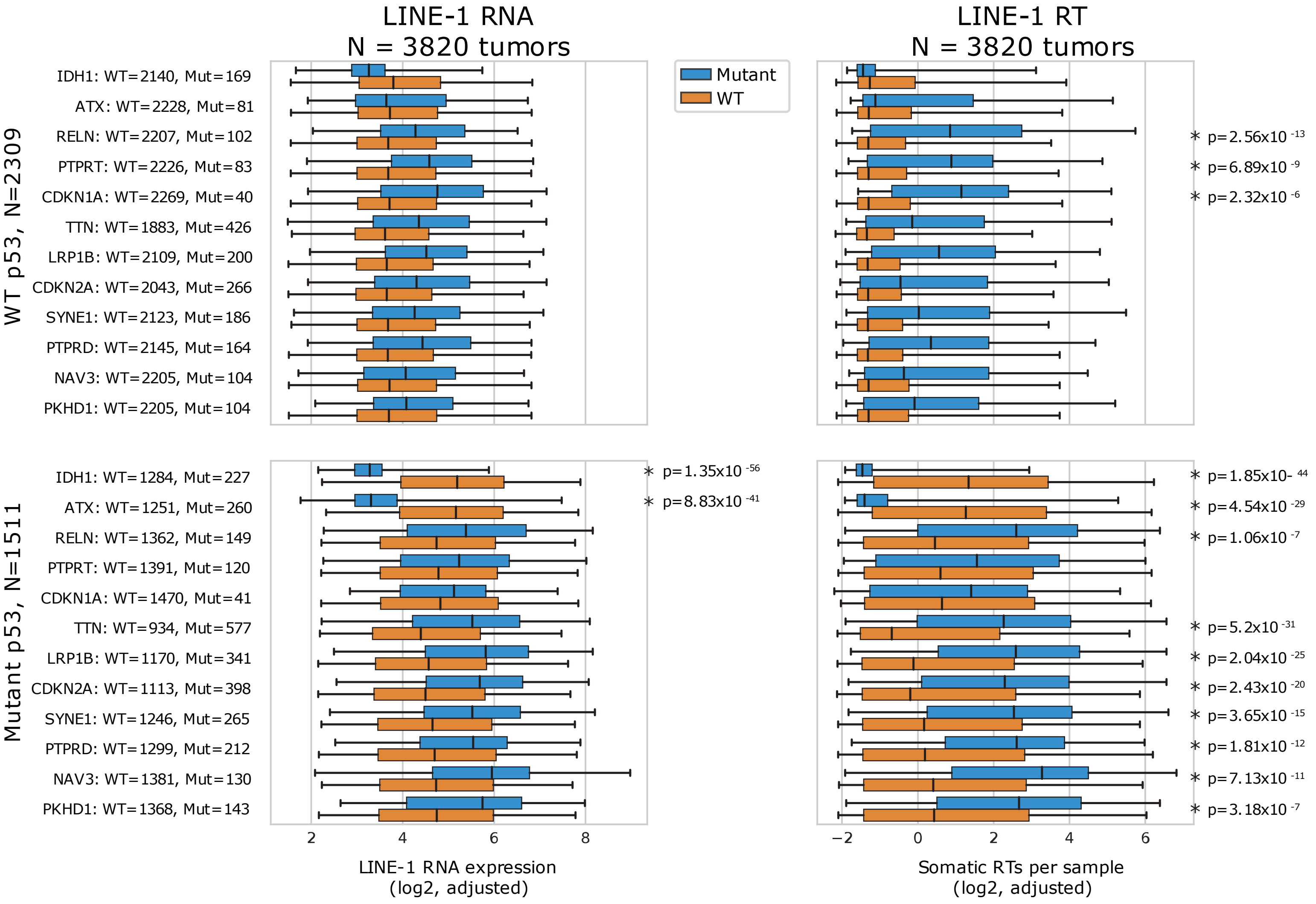

Supplementary Figure 15 | Additional gene mutations may influence L1 activity. Stratifying L1 RNA expression (left, log2 TPM adjusted) and L1 RT burden (right, log2 count adjusted) among p53 WT (top, N = 2,309) and p53 mutant (bottom, N = 1,511) tumors by mutation status in frequently mutated genes. The 82 most frequently mutated genes in TCGA were tested. For each comparison, a two-sided Mann-Whitney U test was evaluated to compare the tumors with mutations in the given gene to tumors without mutations in that gene. * indicates genes where $p < 0.01$ following multiple hypothesis correction (Bonferroni adjustment). All tested genes with any significant comparison are shown here. Blue boxes, samples with mutations in the given gene. Orange boxes, samples that are WT for the given gene. Center line indicates median. Box indicates interquartile range. Whiskers extend to 95% confidence interval; outliers falling outside this range are not shown for clarity. All significant comparisons here had multiple hypothesis-corrected $p < 2 \times 10^{-3}$.

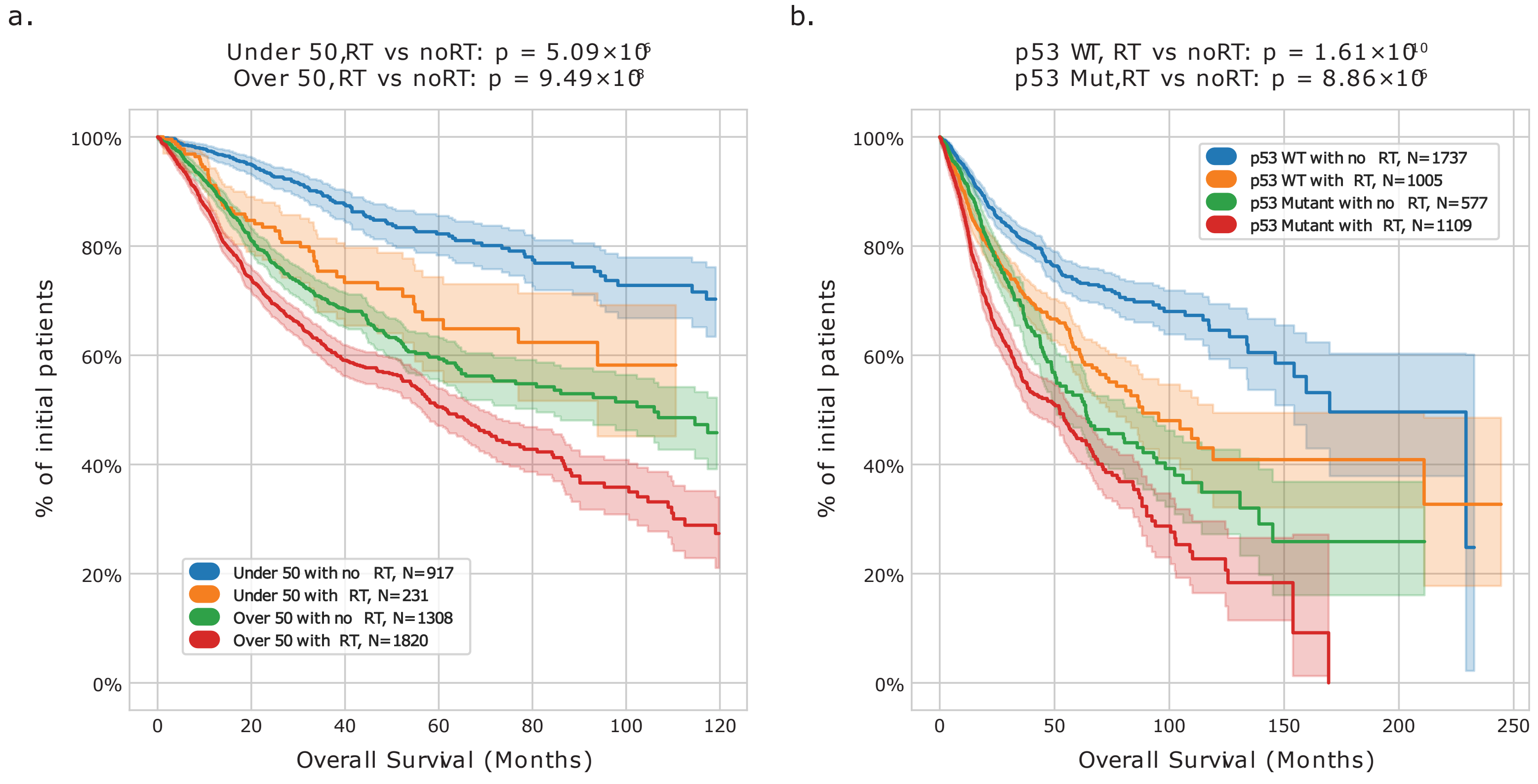

Supplementary Figure 16 | Overall patient survival for those having tumors with or without RT, plotted using Kaplan-Meier method. Shaded areas around curves show 95% confidence intervals. Significance calculated from log-rank test. a) Survival stratified by tumors with RT and diagnosis age under or over 50 years. Diagnosis age classification based on annotations from github.com/GerkeLab/TCGAclinical. Among tumors diagnosed under 50 years, b) Survival stratified by tumors with RT and p53 mutation. p53 mutant or WT classification based on annotations from cBioPortal. See Stratifying dataset by clinical annotations in Supplementary Methods for methodology.

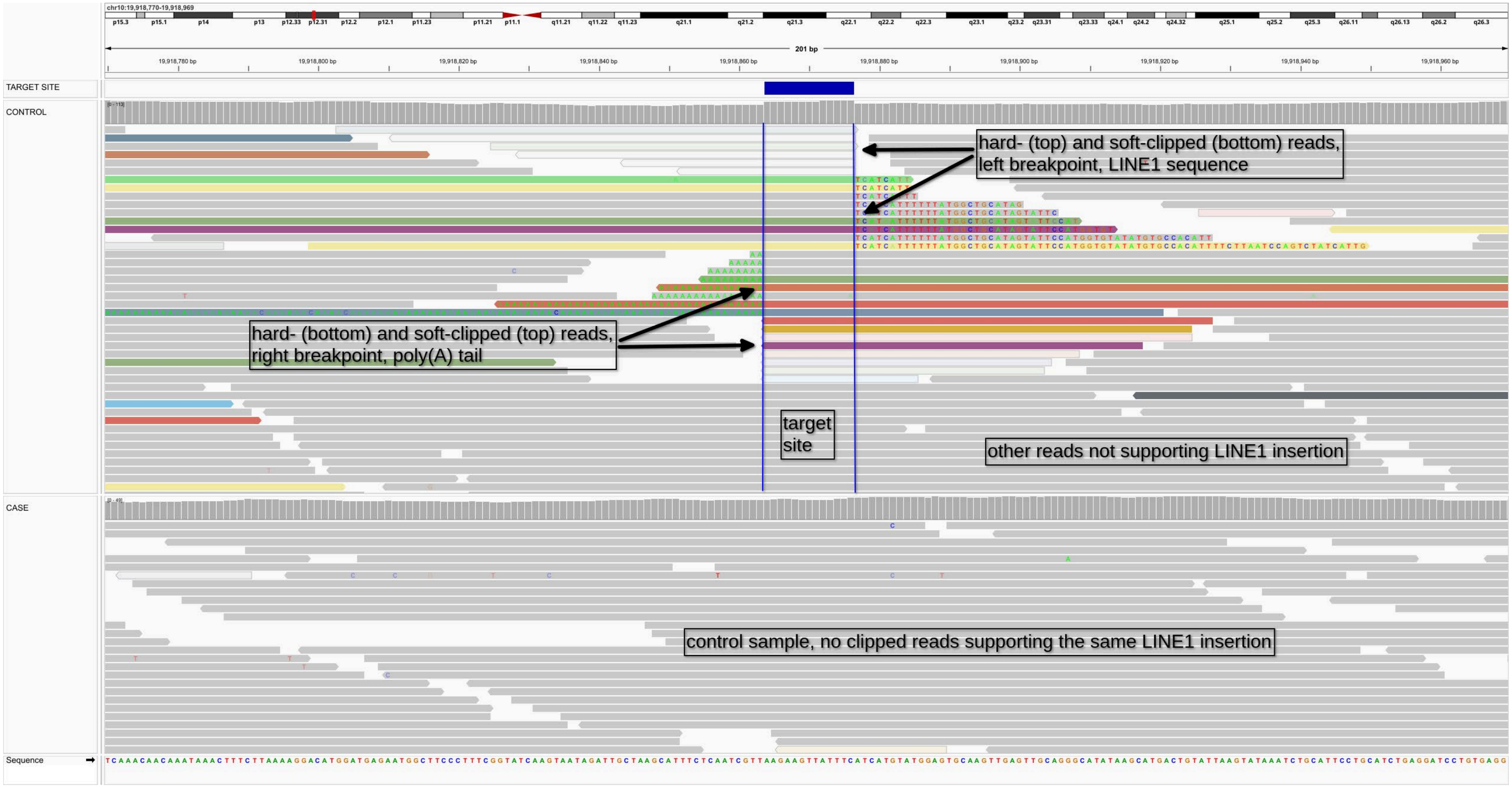

Supplementary Figure 17 | IGV screenshot of a site with a L1 retrotransposition. Possible duplicates and secondary alignments are not shown. TotalReCall identifies 15 clipped reads (9 soft clipped + 6 hard clipped) with matching sequences for the 5' end breakpoint and maps the representative sequence to L1, while xTea identifies 6 clipped reads that can be mapped to L1 individually. Use of hardclipped reads (supplementary alignments, hard clipped bases not shown in IGV) by TotalReCall and inferring the clipped sequence from the matching primary alignments allows it to extend the 3' end of the representative sequence, almost doubling its length from 73 bases (TCATCATTTTTTATGGCTGCATAGTATTCCATGGTGTATATGTGCCACATTTTCTTAATCCAGTCTATCATT G) to 126 bases(TCATCATTTTTT ATGGCTGCATAGTATTCCATGGTGTATATGTGCCACATTTTCTTAATCCAGTCTATCATTGTTGGACATTTGGGTTGGTTCCAAGTCTTTGCTATT GTGAATAGTGCCGCAATA). This sequence maps exactly to the negative strand of L1 consensus sequence indicating that twin priming occurred and this is an inversion-containing retrotransposition.

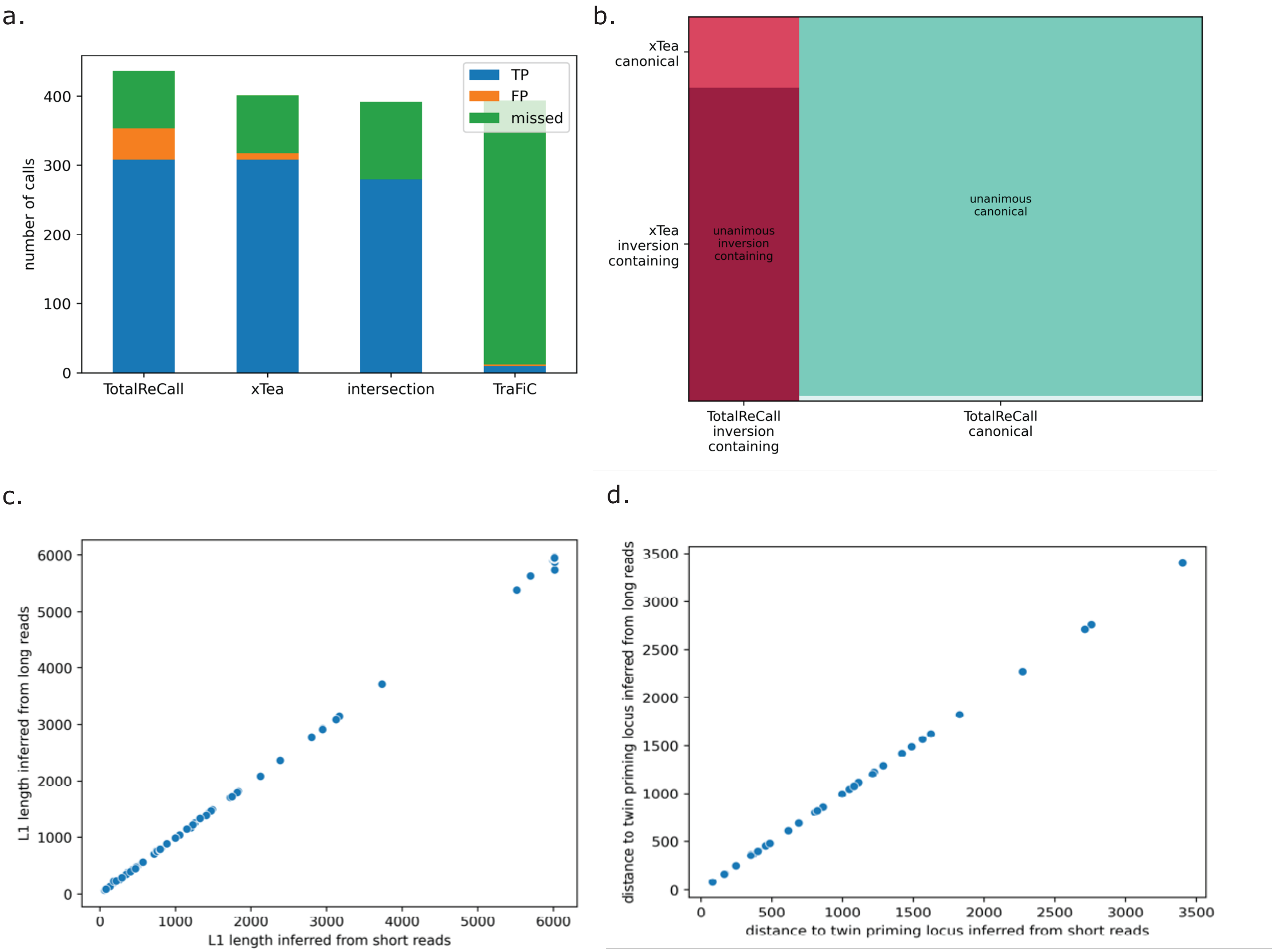

Supplementary Figure 18 | Benchmark data using the GIAB dataset. a) Taking the intersection of TotalReCall and xTea calls removes calls not confirmed by insertions in the long reads. Using TotalReCall or xTea alone results in sensitivity of 0.79 and FDR of 0.13 and 0.03, correspondingly. Using intersection of TotalReCall and xTea calls in this benchmark slightly decreases sensitivity to 0.71 but completely removed false positives (FDR=0). Sensitivity was computed using the insertions present in long reads (those called using any of the three methods for short reads and confirmed by long reads or those called using tldr method1 for long reads directly). b) Most of the calls from the intersection between TotalReCall and xTea agree on the inversion status. We verified the discordant calls by running BLASTn on the inserted sequences obtained directly from alignment of long reads to the genome and confirmed that the TotalReCall inference of inversion was correct (see also Supplementary Table 2). Fraction of inversion-containing L1 insertions in this dataset is consistent with the one determined from "genome archeology."[2] c) There is a very good agreement between the transposon length inferred from the short reads (Illumina, TotalReCall) and the long reads (Oxford Nanopore) ($R > 0.99$, $p < 10^{-10}$, Pearson correlation). Insertion sequences form the long reads were trimmed of possible 3' transductions and the poly(A) tail before computing the transposon length. Only canonical transductions are shown. d) There is a very good agreement between the distance from the 3' end to the locus where the twin priming occurred inferred from the short reads (Illumina, TotalReCall) and the long reads (Oxford Nanopore) ($R > 0.99$, $p < 10^{-10}$, Pearson correlation) for inversion-containing transductions.

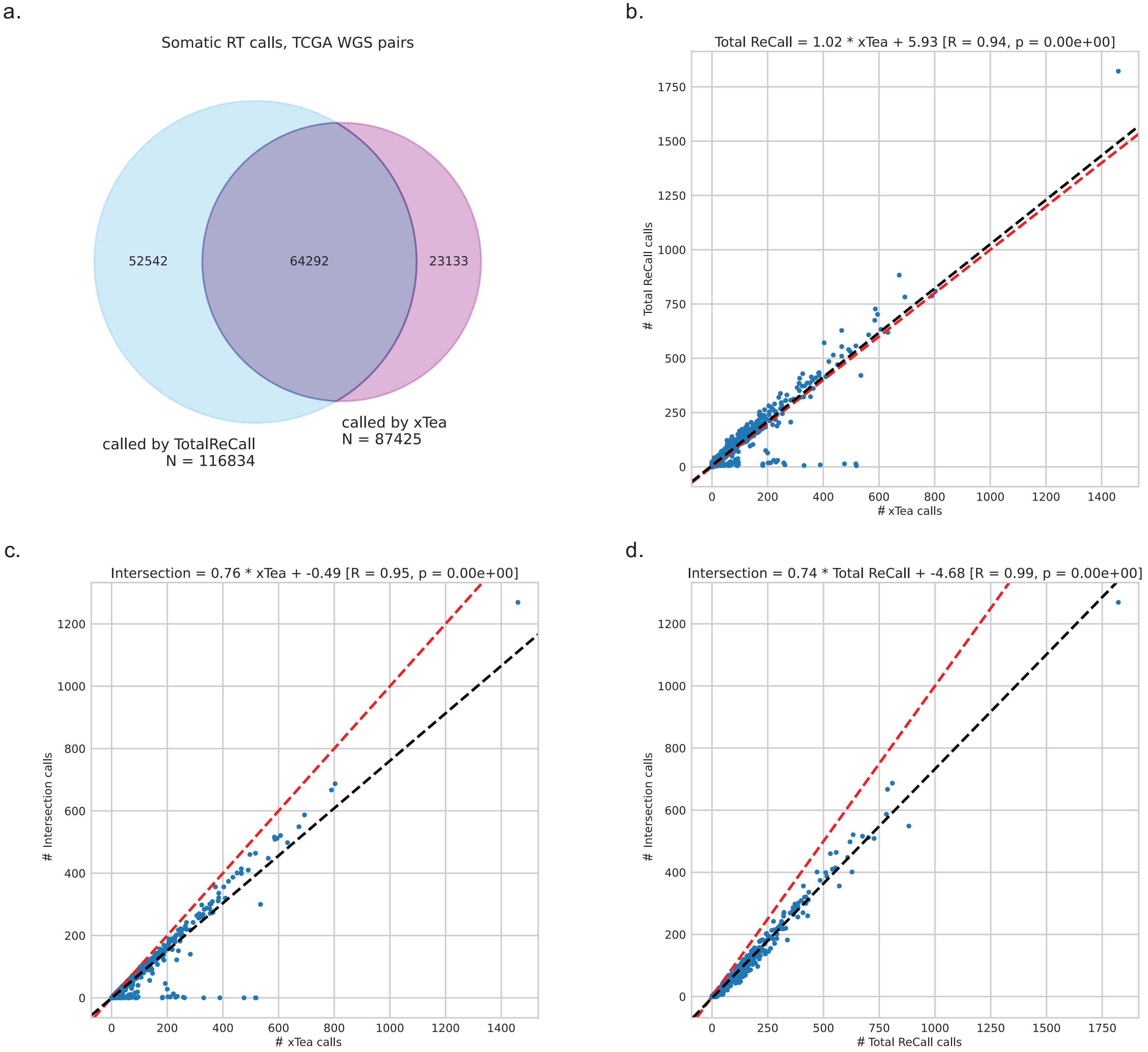

Supplementary Figure 19 | Taking the intersection of RT calls from xTea and TotalReCall. N = 4,669 tumors. a) Venn diagram of total calls throughout dataset from either or both callers. b) Correlation between samplelevel count of calls from TotalReCall (y-axis) vs. xTea (x-axis). R = 0.94, $p < 10^{-10}$, Pearson correlation. c) Correlation between sample-level count of calls from the intersection set (y-axis) vs xTea (x-axis). R = 0.95, $p < 10^{-10}$, Pearson correlation. d) Correlation between sample-level count of calls from the intersection set (y-axis) vs TotalReCall (x-axis). R = 0.99, $p < 10^{-10}$, Pearson correlation. b-d) Red line shows y=x. Black line shows linear regression best fit, ordinary least squares.

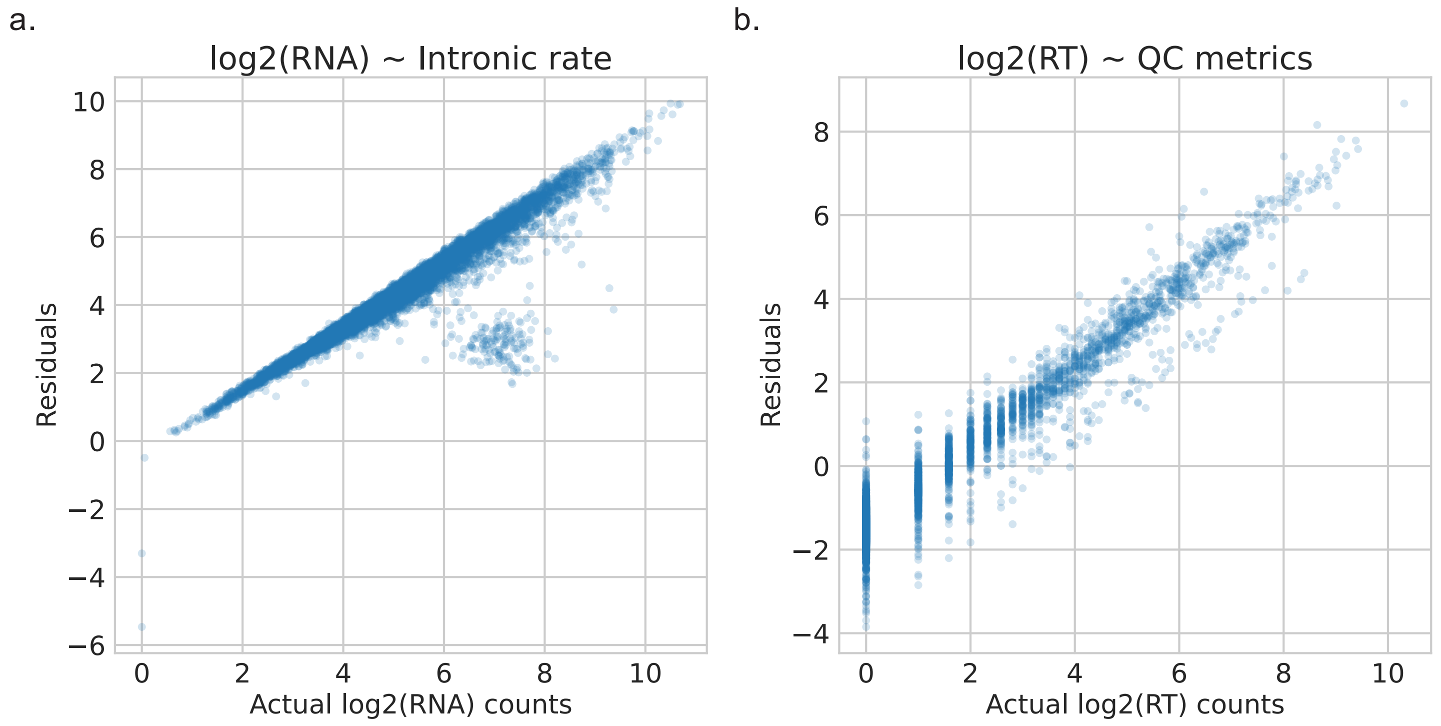

Supplementary Figure 20 | Adjusting L1 RNA and RT measurements. Adjustments based on residuals from linear regression model with QC metrics vs. raw measurements. a) Adjusted estimates of L1 RNA expression per sample (log2 TPM) based on intronic rate of RNA-seq. N = 9,717 tumor and normal samples. b) Adjusted counts of somatic L1 RT per sample (log2 count) based on total coverage, average base quality, read length, rate of clipped bases, and rate of chimeric alignments in both the tumor and paired normal WGS. N = 4,669 tumor samples.

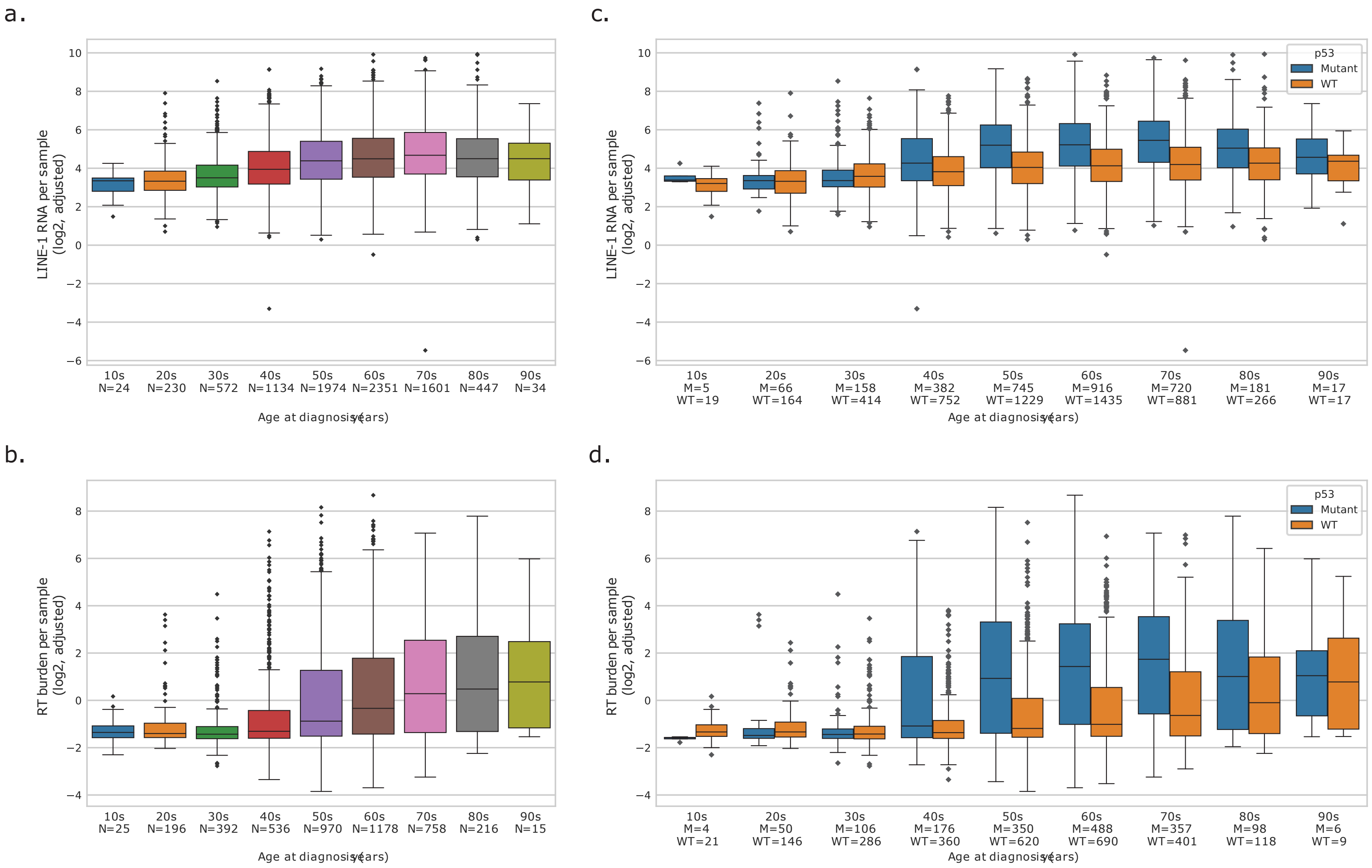

Supplementary Figure 21 | Stratifying L1 RNA and RT by patient age. a) Box plots representing L1 RNA expression (log2 TPM, adjusted) within all samples, stratified by decade of age at diagnosis. Total N = 8,833 tumors for which we have both RNA and diagnosis age data. b) Box plots representing L1 RT burden (log2 count, adjusted) within all samples, stratified by decade of age at diagnosis. Total N = 4,463 tumors for which we have both WGS and diagnosis age data. c) Box plots representing L1 RNA expression (log2 TPM, adjusted) within all samples, stratified by decade of age at diagnosis and p53 mutation. d) Box plots representing L1 RT burden (log2 count, adjusted) within all samples, stratified by decade of age at diagnosis and p53 mutation. a-d) Center lines indicate mean. Boxes indicate interquartile range. Points more than 1.5 x IQR away from the IQR box are shown as individual outliers. Diagnosis age classification based on annotations from github.com/GerkeLab/TCGAclinical. c-d) Blue boxes, tumors with mutant p53. Orange boxes, tumors with WT p53. p53 mutant or WT classification based on annotations from cBioPortal.

Dictionary for Supplementary Data

Supplementary Data	Column	Description
**Supplementary Data 3**	sample_id	TCGA sample ID
	aliquot_id	TCGA aliquot ID
	patient_id	TCGA patient ID
	sample_type	TCGA sample type
	subtype	TCGA tumor subtype abbreviation
	RNA	RNA-seq available
	WGS	WGS available
	p53	p53 mutation status available
	RT_burden	Number of detected retrotranspositions
	RT_burden_log2	Number of detected retrotranspositions, log2
	RT_burden_adj	Number of detected retrotranspositions, adjusted for QC metrics
	RT_burden_log2_adj	Number of detected retrotranspositions, adjusted for QC metrics, log2
	all_loci_TPM	Total LINE-1 TPM
	all_loci_TPM_log2	Total LINE-1 TPM, log2
	all_loci_TPM_adj	Total LINE-1 TPM, adjusted for QC metrics
	all_loci_TPM_log2_adj	Total LINE-1 TPM, adjusted for QC metrics, log2
	active_loci_TPM	LINE-1 TPM across active loci
	active_loci_TPM_log2	LINE-1 TPM across active loci, log2
	active_loci_TPM_adj	LINE-1 TPM across active loci, adjusted for QC metrics
	active_loci_TPM_log2_adj	LINE-1 TPM across active loci, adjusted for QC metrics, log2
	lfs	LFS status
	Intronic Rate	Intronic rate
	TP53	Specific p53 mutation
	binary_*	Boolean indicators for whether 82 genes are mutated in the individual's tumor
	Chimera fraction_T	Quality metrics related to the tumor and paired normal WGS samples used to adjust RT burden estimates
	Clipped bases fraction_T	Quality metrics related to the tumor and paired normal WGS samples used to adjust RT burden estimates
	Average (usable) read length_T	Quality metrics related to the tumor and paired normal WGS samples used to adjust RT burden estimates
	Average (usable) base quality_T	Quality metrics related to the tumor and paired normal WGS samples used to adjust RT burden estimates
	Total number of (usable) aligned bases (coverage)_T	Quality metrics related to the tumor and paired normal WGS samples used to adjust RT burden estimates
	Chimera fraction_N	Quality metrics related to the tumor and paired normal WGS samples used to adjust RT burden estimates
	Clipped bases fraction_N	Quality metrics related to the tumor and paired normal WGS samples used to adjust RT burden estimates

	Average (usable) read length_N	Quality metrics related to the tumor and paired normal WGS samples used to adjust RT burden estimates
	Average (usable) base quality_N	Quality metrics related to the tumor and paired normal WGS samples used to adjust RT burden estimates
	Total number of (usable) aligned bases (coverage)_N	Quality metrics related to the tumor and paired normal WGS samples used to adjust RT burden estimates
**Supplementary Data 6**	Comparison	Comparison identifier
	"Case" sample	Case sample identifier
	"Control" sample	Control sample identifier
	chr	Chromosome of insertion site
	position	Position of insertion site
	TotalReCall	Annotation for inversion or canonical as reported by TotalReCall
	xTea	Annotation for inversion or canonical as reported by xTea
	Truth	True value off inversion or canonical based on BLASTn alignments of long read sequences
	ONT representative sequence 1	Sequences representative of the insertion from Oxford Nanopore reads
	ONT representative sequence 2	Sequences representative of the insertion from Oxford Nanopore reads
	subject	BLASTn alignment output for ONT representative sequence 1
	% identity	BLASTn alignment output for ONT representative sequence 1
	alignment length	BLASTn alignment output for ONT representative sequence 1
	mismatches	BLASTn alignment output for ONT representative sequence 1
	gap opens	BLASTn alignment output for ONT representative sequence 1
	q.start	BLASTn alignment output for ONT representative sequence 1
	q.end	BLASTn alignment output for ONT representative sequence 1
	s.start	BLASTn alignment output for ONT representative sequence 1
	s.end	BLASTn alignment output for ONT representative sequence 1
	evalue	BLASTn alignment output for ONT representative sequence 1
	bitscore	BLASTn alignment output for ONT representative sequence 1
	subject	BLASTn alignment output for ONT representative sequence 2
	% identity	BLASTn alignment output for ONT representative sequence 2
	alignment length	BLASTn alignment output for ONT representative sequence 2
	mismatches	BLASTn alignment output for ONT representative sequence 2
	gap opens	BLASTn alignment output for ONT representative sequence 2

	q.start	BLASTn alignment output for ONT representative sequence 2
	q.end	BLASTn alignment output for ONT representative sequence 2
	s.start	BLASTn alignment output for ONT representative sequence 2
	s.end	BLASTn alignment output for ONT representative sequence 2
	evalue	BLASTn alignment output for ONT representative sequence 2
	bitscore	BLASTn alignment output for ONT representative sequence 2
	Comment	Comment on BLASTn alignments, noting the presence of transductions in some insertions.
**Supplementary Data 7**	SAMPLE_NAME	Analysis sample name
	CHROM	Insertion chromosome location
	POS_TR	Insertion start (TotalReCall)
	END_TR	Insertion end (TotalReCall)
	POS_XT	Insertion start (xTea)
	END_XT	Insertion end (xTea)
	INVERSION_TR	Inversion call (TotalReCall)
	INS_INV_XT	Inversion call (xTea)
	SVLEN_TR	Inferred length of insertion, TotalReCall
	SVLEN_XT	Inferred length of insertion, xTea
	pass_transduction	Insertion annotated with a transduction that passed multi-mapping-based filtering
	source_l1em_locus	Source locus
	TD_SRC	Original transduction annotation, xTea
	BP3_TR	The target site breakpoint associated with the 3' end of the LINE-1 insertion, TotalReCall
	BP3SEQ_TR	The inserted sequence joined to the target site at the 3' end of the LINE-1 insertion, TotalReCall
	BP5_TR	Target site breakpoint associated with the 5' end of the LINE-1 insertion, TotalReCall
	BP5SEQ_TR	Inserted sequence joined to the target site at the 5' end of the LINE-1 insertion, TotalReCall
	TS_SEQUENCE_TR	Sequence of the target site, TotalReCall
	TS_TYPE_TR	Type of alteration of the target site, TotalReCall
	REF_REP_XT	Target site falls within a reference repeat, xTea
	GENE_INFO_XT	Target site falls within a gene, xTea
	sample_id	TCGA sample ID
	dist	Distance (bp) between the totalrecall annotation and xTea annotation

Description of Additional Supplementary Files

Supplementary Data 1
Description: List of unique genomic source loci producing TRTs, and the number of "offspring" they were found to produce in our analysis. Type indicates whether the source locus is a reference or polymorphic copy.

Supplementary Data 2
Description: Listing of N=121 "active" loci used in our analysis, defined as those for which there is evidence of retrotransposition from either our analysis (87 elements) or previously published studies.

Supplementary Data 3
Description: Sample-level summaries of RT, RNA, and gene mutation. A) TCGA sample id. B) TCGA aliquot id, related to WGS sample used, to ensure de-duplication of individuals. C) TCGA patient id. D) Type of sample (Tumor or Normal). E) TCGA study.

Supplementary Data 4
Description: Genome in a Bottle resources. A) Sample name. B) Relationship of this sample within the family trio. C) Sequencing platform. D) File type. E) Link used to access data.

Supplementary Data 5
Description: Complete list of true-positive insertion calls shared by TotalReCall and xTea where the annotations for whether the insertion has an inversion differ between the two callers. A) Identifier for the comparison pair of samples. "DS" indicates the samples have been downsampled from the original depth to approximate 80x coverage in the case sample and 35x coverage in the control sample. B) Sample used as the "case" for a particular comparison. Insertions were unique to this sample when compared against the control. C) Sample used as the "control" for a particular comparison. D-E) Genomic coordinate of insertion site. F-G) Annotation for inversion or canonical as reported by TotalReCall (F) and xTea (G). Highlight color indicates which annotation agrees with the truth value identified by long reads. H) True value of inversion or canonical based on BLASTn alignments of long read sequences. Details in subsequent columns. I-J) Sequences representative of the insertion from Oxford Nanopore reads. K-AF) Results of BLASTn alignments of representative sequences 1 (K-U) and 2 (V-AF) to the L1HS consensus sequence. A single alignment is consistent with a canonical insertion, and two alignments with opposite orientations are consistent with an inversion-containing insertion. In every case of disagreement between TotalReCall and xTea annotations, the long reads supported the TotalReCall annotation. AG) Comment on BLASTn alignments, noting the presence of transductions in some insertions.

Supplementary Data 6
Description: Complete WGS dataset used in this study. A) Unique pair identifier. B) Tumor TCGA sample name. C) Normal TCGA sample name. D) TCGA Project patient belongs to. E-H) DRS URIs used to access tumor and normal alignment files and indices.

Supplementary Data 7
Description: All individual calls from the intersection call set. A) Unique identifier per sample given by the TCGA aliquot ids for the tumor and normal samples used as a pair. B) Chromosome of insertion. C-F) Target site left and right positions, as indicated by totalrecall ("_TR") or xTea ("_XT"). G-H) Presence of an inversion of the LINE-1 sequence within this insertion, consistent with twin-priming. I-J) Inferred length of inserted sequence. Most accurate when an inversion and transduction are not present. K) Whether this insertion is annotated with a transduction that passed multi-mapping-based filtering. L) For insertions with filter-passing transductions, the corresponding name of the source element in L1EM. If a corresponding element does not exist in L1EM, a unique name is defined in order to count shared source elements. M) The original transduction annotation as output by xTea. N) The target site breakpoint associated with the 3' end of the LINE-1 insertion, as annotated by totalrecall. 0) The inserted sequence joined to the target site at the 3' end of the LINE-1 insertion, as annotated by totalrecall. Sequences are oriented 5' to 3' from the target site into the insertion. P) The target site breakpoint associated with the 5' end of the LINE-1 insertion, as annotated by totalrecall. Q) The inserted sequence joined to the target site at the 5' end of the LINE-1 insertion, as annotated by totalrecall. Sequences are oriented 5' to 3' from the target site into the insertion. R) Sequence of the target site, as annotated by totalrecall. S) Type of alteration of the target site (either duplication, "tsdup", or less frequently deletion, "tsdel") as annotated by totalrecall. T) Whether the target site falls within a reference repeat, as annotated by xTea. U) Whether the target site falls within a gene, as annotated by xTea. V) Corresponding TCGA tumor sample id. W) Distance (bp) between the totalrecall annotation and xTea

annotation.

Supplementary Data 8
Description: Complete RNA-seq dataset used in this study. A) TCGA sample name. B) Sample type (tumor or normal). C) TCGA Project patient belongs to. D) Extended sample type (only deduplicated primary tumors and normals were used in the final dataset). E-J) Booleans indicating filtering used to determine final dataset. Only samples with "TRUE" in column J are used in this study. K) DRS URIs used to access alignment files.

Supplementary Data 9
Description: Stratifying LINE-1 RNA and RT burden by 82 frequently mutated genes. For every tumor with WGS, RNA-seq, and mutation data (N = 3820), every gene is either mutated or not mutated as annotated by cBioPortal (Gao et al, 2013). A) Gene symbol of the gene being tested. B-0) Comparisons of the tumors with mutations in the given gene to tumors WT for the given gene throughout the dataset of 3820 tumors. B) N tumors with a mutation in the given gene. C) N tumors wildtype for the given gene. D-I) Comparing LINE-1 RNA expression in the mutant vs wildtype tumors. D-G) Median (D and F) or Mean (E and G) LINE-1 RNA value within the mutant (D-E) or wildtype (F-G) tumors. H-I) P-value (H) and multiple-hypthesis-corrected p-value (I) for two-sided Mann-Whitney U test comparing the mutant and wildtype tumors. J-0) as D-I, but comparing RT burden. P-AC) Repeating all comparisons B-O within the subset of tumors with wildtype p53, N = 2329. AD-AQ) Repeating all comparisons B-O within the subset of tumors with mutant p53, N = 1491.

Supplementary Data 10
Description: Locus-level TRTs and efficiency. A) Unique locus cytoband id. B) Name of LINE-1 element as named by L1EM. C-E) Genomic coordinates of LINE-1 element. F) Cluster this locus was assigned to, as seen in Figure 5. G) Total count of TRTs from this locus identified throughout our dataset of 4,669 tumors. H) Count of unique individuals with TRTs from this locus throughout our dataset of 4,669 tumors. I-P) Related to the efficiency model, where a linear regression is fitted for locus TRT as a function of locus RNA, tumor type, and p53; N = 3,820 tumors. I) Count of TRTs identified from this locus within the subset of 3,820 tumors. J) Fitted coefficient, interpreted as "efficiency" of the locus. K) Standard error of the coefficient fit, as assigned by the OLS regression. L) T-value of the locus coefficient, as assigned by the OLS regression. M) P-value of the locus coefficient, as assigned by the OLS regression. N-O) Lower and upper bounds of 95% confidence interval around the coefficient estimate in (J). P) Category this locus is assigned to, relative to the background distribution, as seen in Figure 6. Q) Boolean indicating whether this locus is among the 637 sequence-resolved LINE-1 loci annotated in Ebert et al, 2021 (Supplemental Table 22). R) Boolean indicating whether this locus is among the 198 known-active full-length L1s annotated in Ebert et al, 2021 (Supplemental Table 23). S) Boolean indicating whether activity of this locus was measured in vitro in Brouha et al, 2003. Loci assayed in that study but for which no data resulted (annotated as "ND" in Supplemental Table 4) are labeled false here. T) Boolean indicating whether TRTs and RNA from this locus were used to generate the background distribution used to evaluate significance of efficiency estimates, as seen in Figure 6 and Extended Data Figure 4. U-AB) Related to the linear regression of Total RT burden as a function of locus RNA as seen in Extended Data Figure 5; N = 3,879 tumors. U) Fitted coefficient assigned to this locus. V) Standard error of the coefficient fit, as assigned by the OLS regression. W) T-value of the locus coefficient, as assigned by the OLS regression. X) P=value of this locus coefficient, as assigned by the OLS regression. Y-Z) Lower and upper bounds of 95% confidence interval around the coefficient estimate in (U). AA) Overall correlation coefficient for the locus-specific OLS regression, Pearson correlation. AB) Category this locus is assigned to, relative to the background distribution. AC-BE) Mean log2 RNA per tumor type, within tumors with both RNA-seq and WGS, N = 3,879. Tumor types abbreviated as standard for TCGA studies. BF-CH) Mean log2 locus TRT per tumor type, within tumors with both RNA-seq and WGS, N = 3,879. Tumor types abbreviated as standard for TCGA studies. For full cancer type names, see https://gdc.cancer.gov/resources-tcga-users/tcga-code-tables/tcga-study-abbreviations. CI-CK) Edit distance (nucleotides) between the reference sequence at each locus and the L1HS consensus sequence. CI) The entire L1HS consensus sequence (6032bp). CJ) The ORF1 region of the L1HS consensus sequence (1017bp). CK) The ORF2 region of the L1HS consensus sequence (3828bp).
