# Supplementary material for: Pan-cancer multi-omic model of LINE-1 activity reveals locus heterogeneity of retrotransposition efficiency
Source: Nat Commun. 2025 Feb 28;16:2049. doi: 10.1038/s41467-025-57271-1 (PMC11871128; doi:10.1038/s41467-025-57271-1)
Supplement: Supplementary file 3 — Reporting Summary [file 41467_2025_57271_MOESM3_ESM.pdf]

nature portfolio

Corresponding author(s): Benjamin Greenbaum, Alexander Solovyov

Last updated by author(s): Dec 24, 2024

Reporting Summary

Nature Portfolio wishes to improve the reproducibility of the work that we publish. This form provides structure for consistency and transparency in reporting. For further information on Nature Portfolio policies, see our Editorial Policies and the Editorial Policy Checklist.

Statistics

For all statistical analyses, confirm that the following items are present in the figure legend, table legend, main text, or Methods section.

n/a	Confirmed	
☐	☒	The exact sample size (*n*) for each experimental group/condition, given as a discrete number and unit of measurement
☒	☐	A statement on whether measurements were taken from distinct samples or whether the same sample was measured repeatedly
☐	☒	The statistical test(s) used AND whether they are one- or two-sided<br>*Only common tests should be described solely by name; describe more complex techniques in the Methods section.*
☐	☒	A description of all covariates tested
☐	☒	A description of any assumptions or corrections, such as tests of normality and adjustment for multiple comparisons
☐	☒	A full description of the statistical parameters including central tendency (e.g. means) or other basic estimates (e.g. regression coefficient) AND variation (e.g. standard deviation) or associated estimates of uncertainty (e.g. confidence intervals)
☐	☒	For null hypothesis testing, the test statistic (e.g. *F*, *t*, *r*) with confidence intervals, effect sizes, degrees of freedom and *P* value noted<br>*Give P values as exact values whenever suitable.*
☒	☐	For Bayesian analysis, information on the choice of priors and Markov chain Monte Carlo settings
☒	☐	For hierarchical and complex designs, identification of the appropriate level for tests and full reporting of outcomes
☐	☒	Estimates of effect sizes (e.g. Cohen's *d*, Pearson's *r*), indicating how they were calculated

Our web collection on statistics for biologists contains articles on many of the points above.

Software and code

Policy information about availability of computer code

Data collection	The data is completely derived from The Cancer Genome Atlas (TCGA).
Data analysis	TotalReCall code for L1 retrotransposition detection from short-read whole-genome sequencing data is publicly available on GitHub (https://github.com/Rome-Tx/totalrecall, https://doi.org/10.5281/zenodo.14553438). The repository includes the source code for the package, a Dockerfile to build the container image, and a WDL file that was used to run TotalReCall using Terra/Cromwell. Pre-built Docker images are published on Dockerhub and publicly available. All downstream analyses were performed in python or R, as specified.

For manuscripts utilizing custom algorithms or software that are central to the research but not yet described in published literature, software must be made available to editors and reviewers. We strongly encourage code deposition in a community repository (e.g. GitHub). See the Nature Portfolio guidelines for submitting code & software for further information.

Data

Policy information about availability of data

All manuscripts must include a data availability statement. This statement should provide the following information, where applicable:

- Accession codes, unique identifiers, or web links for publicly available datasets
- A description of any restrictions on data availability
- For clinical datasets or third party data, please ensure that the statement adheres to our policy

All raw data have been obtained from publicly available datasets, with references cited herein. Specific Genome in a Bottle sequencing data download links are

provided in Supplemental Data 4. Processed data generated in this analysis, in addition to TCGA sequencing data access information, are provided as Supplemental Datas.

Research involving human participants, their data, or biological material

Policy information about studies with human participants or human data. See also policy information about sex, gender (identity/presentation), and sexual orientation and race, ethnicity and racism.

Reporting on sex and gender	We have not collected any new patient data.
Reporting on race, ethnicity, or other socially relevant groupings	We have not collected any new patient data.
Population characteristics	We have not collected any new patient data.
Recruitment	We have not collected any new patient data.
Ethics oversight	We have not collected any new patient data. The use of The Cancer Genome Atlas (TCGA) data does not require IRB approval. For germline TCGA calls, the data have been made available from dbGAP as described in the text.

Note that full information on the approval of the study protocol must also be provided in the manuscript.

Field-specific reporting

Please select the one below that is the best fit for your research. If you are not sure, read the appropriate sections before making your selection.

☒ Life sciences ☐ Behavioural & social sciences ☐ Ecological, evolutionary & environmental sciences

For a reference copy of the document with all sections, see nature.com/documents/nr-reporting-summary-flat.pdf

Life sciences study design

All studies must disclose on these points even when the disclosure is negative.

Sample size	We have used all available TCGA data at the time the study was conducted.
Data exclusions	We have removed low quality whole genome sequencing data where more than 2% of the reads were not properly mapped as a pair.
Replication	The study does not involve experimental work.
Randomization	The study does not involve experimental work.
Blinding	We used the previously collected data from TCGA and GIAB projects.

Reporting for specific materials, systems and methods

We require information from authors about some types of materials, experimental systems and methods used in many studies. Here, indicate whether each material, system or method listed is relevant to your study. If you are not sure if a list item applies to your research, read the appropriate section before selecting a response.

Materials & experimental systems

n/a	Involved in the study	
☒	☐	Antibodies
☒	☐	Eukaryotic cell lines
☒	☐	Palaeontology and archaeology
☒	☐	Animals and other organisms
☒	☐	Clinical data
☒	☐	Dual use research of concern
☒	☐	Plants

Methods

n/a	Involved in the study	
☒	☐	ChIP-seq
☒	☐	Flow cytometry
☒	☐	MRI-based neuroimaging

Plants

Seed stocks	Our study does not use plants.
Novel plant genotypes	Our study does not use plants.
Authentication	Our study does not use plants.
