# Supplementary material for: Pan-cancer multi-omic model of LINE-1 activity reveals locus heterogeneity of retrotransposition efficiency
Source: Nat Commun. 2025 Feb 28;16:2049. doi: 10.1038/s41467-025-57271-1 (PMC11871128; doi:10.1038/s41467-025-57271-1)
Supplement: Supplementary file 4 — Code and Software Submission Checklist [file 41467_2025_57271_MOESM4_ESM.pdf]

nature research

Corresponding author(s): *Double-blind peer review submissions: write DBPR and your manuscript number here instead of author names.*

Code and Software Submission Checklist

Prior to submitting your work to Nature Research, we strongly recommend that you ask at least one colleague who is unfamiliar with your software to install the tool(s), follow the instructions, and provide feedback. This process will help ensure that reviewers will also be able to run your software.

You must submit all required content as a single zip file prior to peer review or provide a link where editors and reviewers can access all required content.

Required content

- [x] Compiled standalone software and/or source code
- [x] A small (simulated or real) dataset to demo the software/code

A README file that includes:

1. System requirements
   - [x] All software dependencies and operating systems (including version numbers)
   - [x] Versions the software has been tested on
   - [x] Any required non-standard hardware
2. Installation guide
   - [x] Instructions
   - [x] Typical install time on a "normal" desktop computer
3. Demo
   - [x] Instructions to run on data
   - [x] Expected output
   - [ ] Expected run time for demo on a "normal" desktop computer
4. Instructions for use
   - [x] How to run the software on your data
   - [ ] (OPTIONAL) Reproduction instructions

     We encourage you to include instructions for reproducing all the quantitative results in the manuscript.

Additional information

Describe your software's license for use. We strongly recommend using a license approved by the Open Source Initiative.

GNU General Public License as published by the Free Software Foundation; either version 2 of the License, or any later version

Provide a link to the code in an open source repository (when available).

https://github.com/Rome-Tx/totalrecall/tree/tcga-paper

Your manuscript should include a complete, detailed description of the code's functionality (i.e. pseudocode).

Please indicate where this is found:

- [ ] Main text
- [ ] Methods section
- [x] Elsewhere (specify):

We provide a DAG of the workflow in the GitHub repository in addition to the Methods section of the manuscript.

Examples of well-structured software packages

1. https://github.com/neurodata-papers/MGC
2. https://github.com/neurodata-papers/LOL
3. https://www.nature.com/nbt/journal/v34/n6/abs/nbt.3569.html#supplementary-information
4. https://www.nature.com/nature/journal/v548/n7669/full/nature23463.html#extended-data
   https://github.com/yasharhezaveh/Ensai
5. https://www.nature.com/nbt/journal/v34/n11/full/nbt.3685.html#supplementary-information
   https://github.com/IFIproteomics/LFQbench
