# Supplementary material for: Pan-cancer multi-omic model of LINE-1 activity reveals locus heterogeneity of retrotransposition efficiency
Source: Nat Commun. 2025 Feb 28;16:2049. doi: 10.1038/s41467-025-57271-1 (PMC11871128; doi:10.1038/s41467-025-57271-1)
Supplement: Supplementary file 5 — Description of Additional Supplementary Files [file 41467_2025_57271_MOESM5_ESM.pdf]

Description of Additional Supplementary Files

Supplementary Data 1
Description: List of unique genomic source loci producing TRTs, and the number of "offspring" they were found to produce in our analysis. Type indicates whether the source locus is a reference or polymorphic copy.

Supplementary Data 2
Description: Listing of N=121 "active" loci used in our analysis, defined as those for which there is evidence of retrotransposition from either our analysis (87 elements) or previously published studies.

Supplementary Data 3
Description: Sample-level summaries of RT, RNA, and gene mutation. A) TCGA sample id. B) TCGA aliquot id, related to WGS sample used, to ensure de-duplication of individuals. C) TCGA patient id. D) Type of sample (Tumor or Normal). E) TCGA study. abbreviation. F-H) Booleans for whether RNA-seq, WGS, and p53 mutation data was available for this study. I-L) Somatic RT burden, either as an exact count, log2-transformed, and/or adjusted using QC metrics. M-P) LINE-1 RNA expression from 1483 L1HS and L1PA2 loci, as calculated by L1EM, either in units TPM, log2 (TPM + 1), and/or adjusted using intronic rate. Q-T) LINE-1 RNA expression from 121 L1HS and L1PA2 loci with observed transductions from this or other studies (Ebert et al, 2021), as calculated by L1EM, either in units TPM, log2 (TPM + 1), and/or adjusted using intronic rate. U) Boolean indicator for whether this patient has a germline p53 mutation. V) Intronic rate used to adjust LINE-1 RNA estimates. W) Specific p53 mutation, as annotated by cBioPortal (Gao et al, 2013). X-DA) Boolean indicators for whether 82 genes are mutated in the individual's tumor, as annotated by cBioPortal (Gao et al, 2013). DB-DK) Quality metrics related to the tumor and paired normal WGS samples used to adjust RT burden estimates.

Supplementary Data 4
Description: Genome in a Bottle resources. A) Sample name. B) Relationship of this sample within the family trio. C) Sequencing platform. D) File type. E) Link used to access data.

Supplementary Data 5
Description: Complete list of true-positive insertion calls shared by TotalReCall and xTea where the annotations for whether the insertion has an inversion differ between the two callers. A) Identifier for the comparison pair of samples. "DS" indicates the samples have been downsampled from the

original depth to approximate 80x coverage in the case sample and 35x coverage in the control sample. B) Sample used as the "case" for a particular comparison. Insertions were unique to this sample when compared against the control. C) Sample used as the "control" for a particular comparison. D-E) Genomic coordinate of insertion site. F-G) Annotation for inversion or canonical as reported by TotalReCall (F) and xTea (G). Highlight color indicates which annotation agrees with the truth value identified by long reads. H) True value of inversion or canonical based on BLASTn alignments of long read sequences. Details in subsequent columns. I-J) Sequences representative of the insertion from Oxford Nanopore reads. K-AF) Results of BLASTn alignments of representative sequences 1 (K-U) and 2 (V-AF) to the L1HS consensus sequence. A single alignment is consistent with a canonical insertion, and two alignments with opposite orientations are consistent with an inversion-containing insertion. In every case of disagreement between TotalReCall and xTea annotations, the long reads supported the TotalReCall annotation. AG) Comment on BLASTn alignments, noting the presence of transductions in some insertions.

Supplementary Data 6

Description: Complete WGS dataset used in this study. A) Unique pair identifier. B) Tumor TCGA sample name. C) Normal TCGA sample name. D) TCGA Project patient belongs to. E-H) DRS URIs used to access tumor and normal alignment files and indices.

Supplementary Data 7

Description: All individual calls from the intersection call set. A) Unique identifier per sample given by the TCGA aliquot ids for the tumor and normal samples used as a pair. B) Chromosome of insertion. C-F) Target site left and right positions, as indicated by totalrecall ("_TR") or xTea ("_XT"). G-H) Presence of an inversion of the LINE-1 sequence within this insertion, consistent with twin-priming. I-J) Inferred length of inserted sequence. Most accurate when an inversion and transduction are not present. K) Whether this insertion is annotated with a transduction that passed multi-mapping-based filtering. L) For insertions with filter-passing transductions, the corresponding name of the source element in L1EM. If a corresponding element does not exist in L1EM, a unique name is defined in order to count shared source elements. M) The original transduction annotation as output by xTea. N) The target site breakpoint associated with the 3' end of the LINE-1 insertion, as annotated by totalrecall. 0) The inserted sequence joined to the target site at the 3' end of the LINE-1 insertion, as annotated by totalrecall. Sequences are oriented 5' to 3' from the target site into the insertion. P) The target site breakpoint associated with the 5' end of the LINE-1 insertion, as annotated by totalrecall. Q) The inserted sequence joined to the target site at the 5' end of the LINE-1 insertion, as annotated by totalrecall. Sequences are oriented 5' to 3' from the target site into the insertion. R) Sequence of the target site, as annotated by totalrecall. S) Type of alteration of the

target site (either duplication, "tsdup", or less frequently deletion, "tsdel") as annotated by totalrecall. T) Whether the target site falls within a reference repeat, as annotated by xTea. U) Whether the target site falls within a gene, as annotated by xTea. V) Corresponding TCGA tumor sample id. W) Distance (bp) between the totalrecall annotation and xTea annotation.

Supplementary Data 8

Description: Complete RNA-seq dataset used in this study. A) TCGA sample name. B) Sample type (tumor or normal). C) TCGA Project patient belongs to. D) Extended sample type (only deduplicated primary tumors and normals were used in the final dataset). E-J) Booleans indicating filtering used to determine final dataset. Only samples with "TRUE" in column J are used in this study. K) DRS URIs used to access alignment files.

Supplementary Data 9

Description: Stratifying LINE-1 RNA and RT burden by 82 frequently mutated genes. For every tumor with WGS, RNA-seq, and mutation data (N = 3820), every gene is either mutated or not mutated as annotated by cBioPortal (Gao et al, 2013). A) Gene symbol of the gene being tested. B-0) Comparisons of the tumors with mutations in the given gene to tumors WT for the given gene throughout the dataset of 3820 tumors. B) N tumors with a mutation in the given gene. C) N tumors wildtype for the given gene. D-I) Comparing LINE-1 RNA expression in the mutant vs wildtype tumors. D-G) Median (D and F) or Mean (E and G) LINE-1 RNA value within the mutant (D-E) or wildtype (F-G) tumors. H-I) P-value (H) and multiple-hypthesis-corrected p-value (I) for two-sided Mann-Whitney U test comparing the mutant and wildtype tumors. J-0) as D-I, but comparing RT burden. P-AC) Repeating all comparisons B-O within the subset of tumors with wildtype p53, N = 2329. AD-AQ) Repeating all comparisons B-O within the subset of tumors with mutant p53, N = 1491.

Supplementary Data 10

Description: Locus-level TRTs and efficiency. A) Unique locus cytoband id. B) Name of LINE-1 element as named by L1EM. C-E) Genomic coordinates of LINE-1 element. F) Cluster this locus was assigned to, as seen in Figure 5. G) Total count of TRTs from this locus identified throughout our dataset of 4,669 tumors. H) Count of unique individuals with TRTs from this locus throughout our dataset of 4,669 tumors. I-P) Related to the efficiency model, where a linear regression is fitted for locus TRT as a function of locus RNA, tumor type, and p53; N = 3,820 tumors. I) Count of TRTs identified from this locus within the subset of 3,820 tumors. J) Fitted coefficient, interpreted as "efficiency" of the locus. K) Standard error of the coefficient fit, as assigned by the OLS regression.

L) T-value of the locus coefficient, as assigned by the OLS regression. M) P-value of the locus coefficient, as assigned by the OLS regression. N-O) Lower and upper bounds of 95% confidence interval around the coefficient estimate in (J). P) Category this locus is assigned to, relative to the background distribution, as seen in Figure 6. Q) Boolean indicating whether this locus is among the 637 sequence-resolved LINE-1 loci annotated in Ebert et al, 2021 (Supplemental Table 22). R) Boolean indicating whether this locus is among the 198 known-active full-length L1s annotated in Ebert et al, 2021 (Supplemental Table 23). S) Boolean indicating whether activity of this locus was measured in vitro in Brouha et al, 2003. Loci assayed in that study but for which no data resulted (annotated as "ND" in Supplemental Table 4) are labeled false here. T) Boolean indicating whether TRTs and RNA from this locus were used to generate the background distribution used to evaluate significance of efficiency estimates, as seen in Figure 6 and Extended Data Figure 4. U-AB) Related to the linear regression of Total RT burden as a function of locus RNA as seen in Extended Data Figure 5; N = 3,879 tumors. U) Fitted coefficient assigned to this locus. V) Standard error of the coefficient fit, as assigned by the OLS regression. W) T-value of the locus coefficient, as assigned by the OLS regression. X) P=value of this locus coefficient, as assigned by the OLS regression. Y-Z) Lower and upper bounds of 95% confidence interval around the coefficient estimate in (U). AA) Overall correlation coefficient for the locus-specific OLS regression, Pearson correlation. AB) Category this locus is assigned to, relative to the background distribution. AC-BE) Mean log2 RNA per tumor type, within tumors with both RNA-seq and WGS, N = 3,879. Tumor types abbreviated as standard for TCGA studies. BF-CH) Mean log2 locus TRT per tumor type, within tumors with both RNA-seq and WGS, N = 3,879. Tumor types abbreviated as standard for TCGA studies. For full cancer type names, see https://gdc.cancer.gov/resources-tcga-users/tcga-code-tables/tcga-study-abbreviations. CI-CK) Edit distance (nucleotides) between the reference sequence at each locus and the L1HS consensus sequence. CI) The entire L1HS consensus sequence (6032bp). CJ) The ORF1 region of the L1HS consensus sequence (1017bp). CK) The ORF2 region of the L1HS consensus sequence (3828bp).
